# Family Mealtimes: A Systematic Umbrella Review of Characteristics, Correlates, Outcomes and Interventions

**DOI:** 10.3390/nu15132841

**Published:** 2023-06-22

**Authors:** Sarah Snuggs, Kate Harvey

**Affiliations:** School of Psychology and Clinical Language Sciences, University of Reading, Reading RG6 6AH, UK; sarah.snuggs@reading.ac.uk

**Keywords:** systematic review, umbrella review, overview of reviews, family meals, children, adolescents, diet, nutrition, weight, psychosocial outcomes

## Abstract

Systematic reviews have examined the multitude of studies investigating family mealtimes and their importance to child/adolescent health and psychosocial outcomes, but the focus of each is limited to specific aspects of family meals (e.g., frequency) and/or specific outcomes (e.g., nutrition). Their findings require synthesis and so a systematic umbrella review was undertaken. Databases were searched to identify systematic reviews (with or without meta-analysis/meta-synthesis) addressing at least one of the following questions: what are the characteristics and/or correlates of family mealtimes; what outcomes are associated with family mealtimes; are interventions aimed at promoting family mealtimes effective? Forty-one eligible reviews were retrieved. Their findings demonstrate that families with children/adolescents typically eat together at least a few days each week. More frequent family meals are predicted by a more positive mealtime environment, more positive attitudes towards family meals, the presence of younger children, and families having more time. Greater family meal frequency protects children/adolescents against a poorer diet, obesity, risk behaviours, poorer mental health and wellbeing, and poorer academic outcomes. Findings from interventions seeking to promote family mealtimes are mixed. This umbrella review provides a comprehensive and integrated understanding of research into family mealtimes, establishing where evidence is sound and where further research is needed.

## 1. Introduction

Over the past fifteen years, family mealtimes have been the focus of extensive research. Studies have examined characteristics of family mealtimes, for example, their frequency or location [1]; their correlates, such as the age of children or parental employment [2]; a range of outcomes they may predict, for example, nutrition [3], weight [4], academic achievement [5], or adolescent risk-taking [6]; and, given their association with positive outcomes, the efficacy of interventions aimed at promoting them [7].

Across studies, definitions of pfamily mealtimes, or family meals, have varied in their specificity. At their simplest, they have been defined in terms of who shares a meal, for example, *meals eaten with the family* [8] or “the act of eating simultaneously with family members” [9]. Some definitions are more specific about the family member present during the meal, for example, *food eaten together with other family members, usually with one adult present* [10]. Other definitions add elements such as location: *a minimum of a child eating a meal with at least one other individual at home* [11] or commensality *a social moment in the day during when food is eaten together with at least one family member* [12]. The most specific definitions refer to several characteristics of family mealtimes, for example, *occasions at set-times of day when most, if not all members of the immediate family eat food together* [13] or *those occasions when food is eaten simultaneously in the same location by more than one family member* [2]. Nevertheless, although the specificities of family mealtime definitions vary, there is agreement about their core features.

This broad literature has been the subject of several systematic reviews with or without meta-analysis/meta-synthesis (referred to henceforth as systematic reviews). The use of rigorous, replicable, systematic approaches to reviewing this research has been successful in providing comprehensive, high-quality evidence. However, the systematic reviews that were undertaken have typically been limited in scope, for example, systematic reviews examining the association between a single characteristic of family mealtimes (e.g., frequency) and a single outcome (e.g., nutrition). Moreover, the volume of evidence produced is substantial and in some areas inconsistent, extends across several disciplines and is rapidly expanding. Given how challenging it is for this body of evidence to be assimilated, an umbrella review that compares, evaluates and synthesises findings is warranted [14].

The purpose of this umbrella review is to integrate the findings of the numerous systematic reviews investigating family mealtimes. It aims to address the following review questions:

1. What are the characteristics of family mealtimes?
2. What are the correlates of family mealtimes?
3. What outcomes, if any, are family mealtimes associated with?
4. Are interventions aimed at promoting family mealtimes effective?

By providing a comprehensive overview of research relating to family mealtimes, this umbrella review will identify areas where evidence is sound and further research not required, along with areas where evidence is conflicting or limited that could be informed by further research.

## 2. Materials and Methods

This umbrella review was pre-registered in PROSPERO (Registration Number: CRD42023414087) and can be accessed at https://www.crd.york.ac.uk/prospero/, accessed on 19 June 2023. The methods deviated in two ways from the pre-registered protocol. Firstly, it was anticipated that both the revised Measurement Tool to Assess Systematic Reviews (AMSTAR-2) [15] and the Risk of Bias Assessment Tool for Systematic Reviews (ROBIS) [16] would be used, but only AMSTAR-2 was used once it had been established that it includes a risk of bias assessment. Secondly, time constraints meant that a second reviewer did not check the first reviewer’s AMSTAR-2 assessments.

The review was conducted in accordance with the Cochrane Collaboration guidelines [17] and reported using the Preferred Reporting Items for Overviews of Reviews (PRIOR) checklist [18], which is similar to the Preferred Reporting Items for Systematic Reviews and Meta-Analysis (PRISMA) [19] but designed for overviews of reviews.

### 2.1. Eligibility Criteria and Information Source

Inclusion and exclusion aligning to Population, Intervention, Comparison and Outcomes (PICO) [20] were agreed in advance by reviewers. Primarily the inclusion criteria reflected the aims of the umbrella review: to ensure full coverage of the literature, systematic reviews with both meta-analysis and meta-synthesis were included, along with narrative reviews. In addition, publication in peer-reviewed journals was used as a proxy for quality, and limited resources meant only reviews published in English were included. Studies were eligible if they met the following inclusion criteria:

- Systematic reviews with or without meta-analysis and/or meta-synthesis;
- Systematic reviews of studies whose participants included families comprising children and/or adolescents aged from birth to 18 years (PICO Population);
- Systematic reviews that reported at least one aspect of family mealtimes (characteristics, correlates, outcomes or interventions) (PICO Intervention, Comparison and Outcome);
- Peer-reviewed;
- English Language.

The following exclusion criteria were applied:

- Systematic reviews of studies whose participants were restricted to adults aged >18 years;
- Systematic reviews of studies in which only non-family meals were investigated e.g., eating at school;
- Systematic reviews of studies investigating breakfast consumption if the focus was exclusively on the nutrition associated with consuming breakfast rather than breakfast as a family mealtime;
- Systematic reviews of studies whose participants were families with children/adolescents with a medical condition that may affect their eating, for example, Type 1 or Type 2 diabetes; eating disorders (e.g., anorexia); feeding or food intake disorders or difficulties; Autism Spectrum Disorder (ASD); Attention Deficit Hyperactivity Disorder (ADHD);
- Systematic reviews of studies where participants were restricted to adolescents/children who were overweight;
- Systematic reviews of studies in which participants were restricted to parents with eating disorders;
- Primary studies reporting original data;
- Non-systematic reviews e.g., scoping reviews;
- Commentaries, editorials, position papers.

The following databases were searched on 29 and 30 April 2023: PsycInfo, PROSPERO; PubMed (MEDLINE); The Cochrane Library (Cochrane Database of Systematic Reviews); Scopus; Web of Science (science and social science citations). The reference lists of obtained articles were hand-searched for additional systematic reviews that met the eligibility criteria. The search was completed on 9 May 2023.

### 2.2. Search Selection

A preliminary search of PsycInfo and PROSPERO was conducted in April 2023 using terms based on those used by Middleton et al. [13]. This preliminary search indicated the need for the following refinements:

- Exclusion of systematic reviews that focused on families with children/adolescents with a medical condition that may affect their eating (see exclusion criteria for detail);
- The inclusion of “review” as a search term in the title but not in the abstract; doing so resulted in the inclusion of articles that described reviewing the literature but were not reviews. Meta-analysis and meta-synthesis were searched for in both the title and the abstract.

Key search terms, supplemented with an asterisk (or other appropriate syntax) to identify multiple forms of the word (e.g., child, children), were combined using the AND/OR operators for the *population* (family* OR families OR parent* OR mother* OR father* OR dad* OR mum* OR mom* OR child* OR adolescen* OR youth), *intervention* (meal* OR dinner*) and *design* (review*. ti OR meta-analy*.ti,ab OR meta-synth*.ti,ab). The only limitation applied was English Language. The search strategy was initially run in PsychInfo and was then adjusted for each database for controlled vocabulary, appropriate syntax and MeSH terms.

De-duplication, title and abstract screening was undertaken by one reviewer. Articles identified for full-text review were assessed independently by two reviewers (KH and SS). Disagreements were discussed until consensus was reached.

### 2.3. Data Extraction

The nature of this umbrella review meant that a wide range of constructs were evaluated. Therefore, along with conventionally extracted study characteristics, the way in which systematic reviews (or original studies) conceptualised, defined and/or operationalised family mealtimes and other relevant constructs was also extracted to provide context for the evaluation and synthesis. Microsoft Excel was used to record and organise extracted data.

The following data were extracted by one reviewer (KH) and checked by a second (SS):

- Authors and year of publication;
- Key search criteria: dates of review; age of participants; designs included/excluded;
- Systematic review question(s);
- Conceptualisation, definition and/or operationalisation of family mealtimes and other constructs relevant to the article;
- Key systematic review characteristics: design; number of articles/studies; quality;
- Key findings and limitations relevant to family mealtime research.

To manage overlap, where systematic reviews included other systematic reviews, the more recent systematic review was scrutinised to determine the extent to which findings from the original review were fully reflected. If findings relating to family mealtimes were reported in full, only the more recent review was considered further. Conversely, if findings relating to family mealtimes were not fully reflected in the more recent review, the original review continued to be considered. Assessing the extent of overlap of original studies included the systematic reviews was considered but judged to be unfeasible given the size and complexity of the task.

### 2.4. Assessment of Quality and Risk of Bias

The revised Measurement Tool to Assess Systematic Reviews (AMSTAR-2) [16] was used to evaluate the included systematic reviews. AMSTAR-2 was selected because it includes an assessment of risk of bias appropriate for this umbrella review. It is a comprehensive, widely used instrument that enables overall confidence in the results of a systematic review to be rated [21]. AMSTAR-2 is not designed to generate an overall score and researchers are guided to make an overall rating that accounts for flaws in critical domains. In this umbrella review, the quality and risk of bias of systematic reviews was categorised as good (4–5 critical flaws); fair (6–8 critical flaws), and poor (9–11 critical flaws).

### 2.5. Data Synthesis

Characteristics of systematic reviews were described, and extracted data reported. Findings were then summarised in relation to the construct(s) that the systematic review assessed:

- Characteristics of family mealtimes;
- Correlates of family mealtimes;
- Outcomes predicted by family mealtimes;
- Interventions aimed at promoting family mealtimes.

Findings relating to each construct were evaluated and synthesised, and heterogeneity was discussed. Where systematic reviews addressed more than one construct, its findings were synthesised and evaluated in each relevant section.

## 3. Results

### 3.1. Search Results

**Figure 1:**
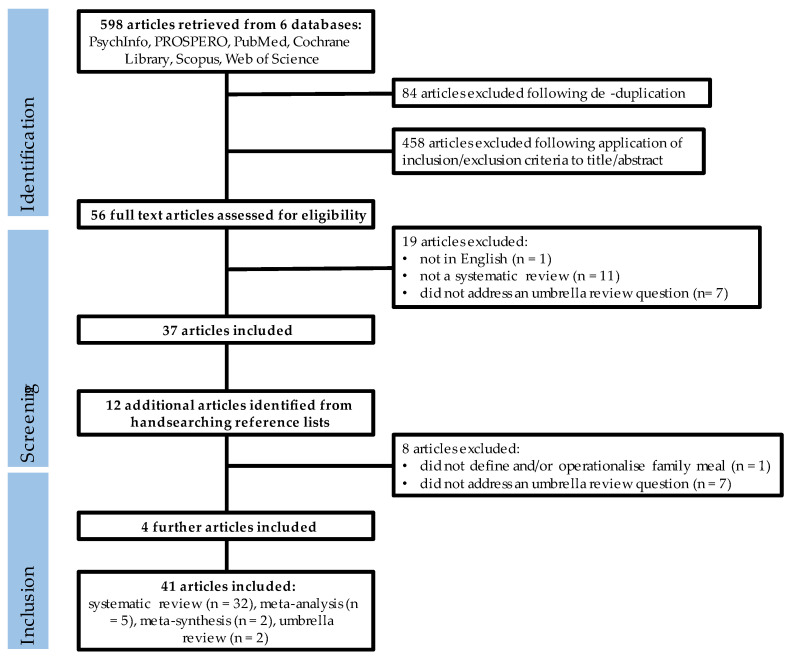
PRISMA flow diagram showing article identification, retrieval and inclusion.

A Preferred Reporting Items for Systematic Review and Meta-Analysis (PRISMA) [19] flow chart details each step in the search process, including the reasons articles were excluded (Figure 1). Supplementary Table S1 details the articles excluded following full-text screening.

The search terms retrieved 598 articles from the six databases. Following de-duplication, the titles and abstracts of 458 articles were screened against the predetermined eligibility criteria (KH). Of the screened articles, the full text of 56 was assessed against the predetermined eligibility criteria (KH and SS independently). Of the 56 assessed articles, 37 were included. Hand-searching the reference lists of these articles identified 12 additional articles for potential inclusion. These articles were screened by two reviewers (KH, SS); seven did not meet the eligibility criteria and one did not define or operationalise “meals”, making it unclear whether meals eaten alone were included. Four articles were included, resulting in a total of 41 articles. Seven of the included systematic reviews did not describe themselves as systematic reviews. Each was reviewed by both KH and SS to establish that, despite not describing themselves as systematic reviews or being registered with PROSPERO, a systematic approach to reviewing the literature that reflected a PROSPERO approach was taken.

### 3.2. Characteristics of the Included Systematic Reviews

Characteristics of the included systematic reviews are shown in Table 1. All were conducted between 2005 and 2022, with the majority (30) having been conducted in the past decade. Systematic reviews of interventions tended to be conducted most recently, while reviews investigating family mealtimes as a predictor of outcome had the broadest range (2005–2022). More than half of the included systematic reviews (24) set no limitations in relation to the date the original articles were published or limited the date to after the 1980s/1990s (Supplementary Table S2).

Full details of the search criteria are reported in Supplementary Table S2. In summary, participants in the original studies always reviewed included children, adolescents and young adults. Most included children/adolescents from birth or aged 2 years to ~18 years (20). Six focused on older children aged ~10–18 years; three focused on younger children with ages ranging from ~birth to 12 years; and one on young children with ages ranging from birth to 3 years. The remaining reviews did not specify the age ranges of participants, describing them variously as children/youth (1), children/adolescents (7) or children/adolescents/young adults (3). Around half (22) specified that original articles should be in peer-reviewed journals; all except three limited original studies to the English language; and all but one excluded studies that included participants with eating disorders. Designs included varied across systematic reviews, but most excluded editorials and commentaries.

Table 1 shows that a majority of the included systematic reviews were narrative; five included a meta-analysis; three a meta-synthesis; and two were umbrella reviews (both investigating the association between family mealtimes and weight/obesity). The number of original articles included by the systematic reviews ranged from 10 to 98 (plus one that included approximately 200 original articles), with a median of 32 original articles. Systematic reviews of interventions to promote family mealtimes tended to include fewer articles, evaluating between two and nine interventions. Six systematic reviews distinguished between the number of original articles that were included and the number of unique datasets.

Details about the way in which family mealtime and other key constructs were conceptualised, defined and/or operationalized are given in Supplementary Table S3. In summary, 10 of the included systematic reviews did not conceptualise, define and/or operationalise family mealtimes. The remaining reviews typically conceptualised family mealtimes as socio-cultural events. Definitions ranged from simply a shared meal eaten with the family through to more complex definitions specifying location, meal type (e.g., dinner) and the presence of specific family members. Family mealtimes were most commonly operationalised as the frequency of family meals, although a handful of studies assessed additional characteristics, for example, mealtime routines, location, duration and the presence of family members.

#### 3.2.1. Constructs Assessed

The majority of systematic reviews (33) investigated the association between family mealtimes and a health or psychosocial outcome. Some described characteristics of family mealtimes (11), some examined correlates of family mealtimes (18) and a few examined interventions aiming to promote family mealtimes (4) (Table 2).

**Figure 2:**
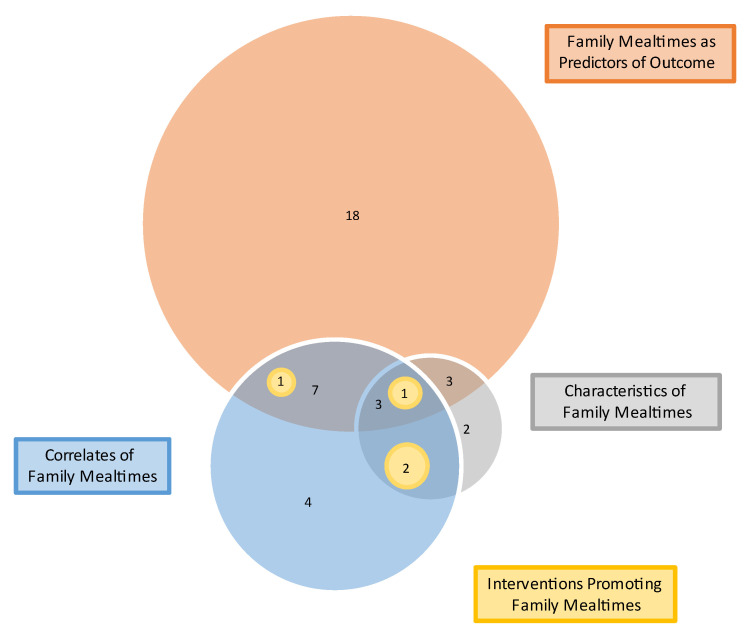
Ven diagram showing constructs assessed by systematic reviews (n = 41).

Figure 2 illustrates that the majority of reviews (24) assessed a single construct, 10 assessed two constructs and 7 assessed three constructs. None assessed all four constructs.

#### 3.2.2. Overlap

Five systematic reviews [2,22,23,24,25] were included in three later systematic reviews [3,8,26] (Supplementary Table S4). The three later reviews were scrutinised to determine the extent to which findings from the original reviews relating to family mealtimes were fully reported. In the case of one original review [6], the findings were fully reported so, to avoid overlap, only the more recent systematic review reporting its findings was considered in the synthesis [3]. Findings relating to family mealtimes in four original reviews [2,22,23,25] were not fully reflected in the more recent reviews that included them [3,8,26] and so continued to be considered independently in the synthesis.

#### 3.2.3. Quality and Risk of Bias

AMSTAR-2 scores (Supplementary Table S5) indicated that the quality of reviews was variable, with reviews having a median of 8 critical flaws (range 4 and 11); eight were categorised as good (4–5 critical flaws); twenty were categorised as fair (6–8 critical flaws), and thirteen as poor (9–11 critical flaws).

- All reviews described their research question and inclusion criteria;
- While some reviews were pre-registered (6) or explicitly stated the review methods were established in advance (11), most had not pre-registered or made an explicit statement (24);
- Few reviews (3) explained their rationale for their selection of study designs;
- In almost all reviews, at least two databases (40) were searched and most reported keywords (37); however, few reviews provided a full rationale for their search limitations (34). In total, only 5 reviews described their literature search strategy comprehensively;
- Approximately half (19) of reviews described at least two reviewers independently agreeing on eligible articles, and approximately one third (15) described at least two reviewers agreeing on the extracted data;
- None of the reviews provided a list of excluded studies or justified exclusions;
- A majority of reviews described the studies included in partial (11) or full (19);
- Reviews that assessed the risk of bias of the original studies also accounted for risk of bias when interpreting their results; however, most reviews (22) did not describe a satisfactory technique for assessing the risk of bias in original studies;
- Most reviews discussed any heterogeneity in the results;
- None of the reviews reported the sources of funding for the original studies that were included;
- A majority of reviews (29) reported potential sources of conflict (including funding); however, reporting may be an artifact of the journal in which the review was published as some require this to be reported while others do not. Of the fourteen that received funding, ten received institutional grants or scholarships, three received industry grants and one did not specify.

Four meta-analyses were included in this umbrella review [11,22,27,28]; all investigated the association between family meals and nutrition/weight. All assessed risk of bias in the individual original studies and used appropriate methods for the statistical combination of results. All established and discussed publication bias.

### 3.3. Characteristics of Family Mealtimes

Eleven systematic reviews investigated the characteristics of family mealtimes.

Definitions of family mealtimes varied widely across original studies [2,8] and the most commonly assessed characteristic, family mealtime frequency, was operationalised in different ways, [22], for example, the frequency of specific meals or the number of shared meals per week, making comparisons difficult.

Approximately from one quarter to one half of families were found to share meals several days each week, one third shared meals a few days each week, and a small proportion (approximately one tenth) did not share meals together [9,10,22,29]. Children typically participated in family meals from about three years old [12].

Studies examining other family mealtime characteristics found most were prepared at home [8], with time, resources and schedules influencing the food that was served [13]. Family meals often took place in rooms other than the kitchen/dining room, the TV was frequently on, and many mothers were not present for the duration of the meal (these studies did not define family meals in relation to family members who were present so may have been restricted to siblings) [10,12]. Difficult and/or disruptive behaviour during family meals was common [13]. Systematic reviews that included fathers found roles in relation to family mealtimes (e.g., cooking and grocery shopping) were shared with mothers [30].

Findings relating to parents’ experiences of family mealtimes revealed that parents hold different ideas about what constitutes a family meal and the extent to which they should be prioritised [13]. Motivations for family meals included health, modelling, the provision of structure/routine, communication, strengthening interpersonal relationships and socialising [1,7,12]. Most original studies sampled mothers, but systematic reviews of original studies focusing on fathers found that, like mothers, fathers are motivated by the opportunity that family meals present to promote healthy eating via modelling, to enforce rules around food and to encourage consumption [30] via a range of strategies [31]. While parents were found to enjoy and value family meals, they also described them as stressful and unpleasant, feeling under pressure to provide them [32]. The stress parents experienced was typically related to children’s picky eating, mess, and difficult/disruptive behaviours during mealtimes [2,12].

### 3.4. Correlates of Family Mealtimes

Eighteen systematic reviews reported data relating to correlates of family mealtime characteristics. Almost all examined correlates of family mealtime frequency, with three examining other correlates, specifically family meal duration, location (eating at the table), planning and food preparation.

#### 3.4.1. Correlates of Family Meal Frequency

Systematic reviews found that more frequent family meals were associated both with children being younger [1,10,29] and with having younger children in the family [32]. Parents being married was found to be associated with more frequent family meals [2,3] and, similarly, single-mother households were found to have family meals less frequently [4,33]. In one of the few systematic reviews of fathers’ behaviours in relation to family mealtimes, Khandpur et al. [34] found that fathers ate with children less frequently than mothers.

Many systematic reviews investigated ethnicity. Findings were often mixed [3], although Martin-Biggers et al. [2] found that Asian-American/Hispanic families had more frequent family meals than African-American families, and Verhage [12] found that Hispanic/non-Hispanic white families had more frequent family meals compared to non-Hispanic black families. Where culture was considered, family meals were found to be more common in Spain and Canada than in the UK and USA [29].

Findings relating to parents’ socio-economic status indicated that parents of a higher socio-economic status had more frequent family meals [2], but findings regarding parents’ education were less clear, with Martin-Biggers et al. [2] concluding that more educated parents had more frequent family meals but Dwyer et al. [7] concluding that the evidence was mixed, with some studies finding positive associations between parents’ education and family meals, others finding no significant association, and others finding positive associations for only some sub-groups.

Systematic reviews found that several characteristics of the family meal influenced their frequency. Eating in the kitchen/dining room without the TV on was associated with more frequent family meals, as was eating with others [35]. On the other hand, adolescents’ desire for autonomy over food eaten was found to negatively impact their likelihood of having family meals [10].

Other aspects of the family environment were also found to be associated with meal frequency; specifically, meals were more frequent in families that prioritised them [7,35], were more positive towards them [7], and where there was better family functioning [11]. Where family relations were poorer, the frequency of family meals declined [10,32]. In terms of food parenting, family meals were more frequent among parents who adopted an authoritative style [2,36].

Some systematic reviews identified characteristics associated with less frequent family meals, namely parents being employed outside the home [2,37] and busy schedules (parents and adolescents) [10]. Consistent with these findings, parents described the lack of resources (time, effort, confidence), planning and mealtime routine as barriers, along with the need to accommodate different family members’ schedules and preferences, and the challenge of getting young children to sit for a meal [2,13].

The COVID-19 pandemic impacted how many families ate together, and the findings align with previous reviews showing that time is important to family meal frequency. Titis [38] examined the specific impact of the restrictions arising from the COVID-19 pandemic on family mealtimes; for many, these restrictions meant confinement at home. Broadly, the studies they reviewed found that, during periods of restriction, parents had more time, a greater interest in health and nutrition, and increased motivation to eat more healthily. This resulted in increased meal-planning and home-cooked meals that were more varied, complex and healthy (in higher-income and dual-parent families). Parents reported eating more family meals, involving children in their preparation more, and a more positive mealtime environment.

While the conclusions of systematic reviews were broadly consistent, two [3,7] findings were notably different. The original studies they examined investigated an extensive range of socio-demographic characteristics in relation to family meal frequency. While some findings were consistent with other systematic reviews, others did not align, with Dwyer et al. [7] finding mixed evidence for children’s sex, parents’ age, parents’ marital status, parents’ education, number of children in the household, parents’ employment, and urban versus rural location, and Glanz et al. [3] finding mixed evidence for ethnicity, parent/child gender, parent/child age, socio-economic status, and education. Why the findings of these reviews were inconsistent, given that they covered a similar time period and population, cannot be established with certainty; however, while the majority of reviews did not define family meals, or restricted their operationalisation of family meals to meal frequency, Dwyer [7] focused on the frequency of *shared meals between parents/caregivers and children* and Glanz [3] on the frequency of *in-home meals that were not necessarily shared,* which may go some way to explaining the heterogeneity of their findings.

#### 3.4.2. Correlates of Other (Non-Frequency) Aspects of Family Meals

While most original studies reviewed operationalised family mealtimes as meal frequency, a few examined other aspects. McCullough et al. [1] examined correlates of family meal duration and location and found a longer duration was associated with adolescents in the family, while eating at the table was associated with children in the family. Jenkins and Horner [37] considered meal-planning, preparation and food served, and found that mothers’ employment outside the home and parents’ work schedules resulted in there being less time for these activities, consistent with Middleton et al.’s [13] finding that the food that is served is influenced by parents’ time, resources and schedules.

### 3.5. Outcomes Associated with Family Mealtimes

Data regarding the outcomes that family mealtimes predicted were reported by 33 systematic reviews. Most examined the association between family mealtime characteristics and health outcomes; because the findings of one [6] were reported in the later review by Glanz et al. [3], to avoid overlap, only Glanz et al. is referred to in this synthesis.

#### 3.5.1. Family Mealtimes as a Predictor of Eating Behaviours/Nutrition

Twenty-eight reviews examined the association between family mealtime characteristics and eating behaviour/nutrition. Eating behaviour/nutrition was operationalised as the consumption of specific meals (typically breakfast), foods (typically fruit, vegetables, dairy, fast-food), macronutrients, and micro-nutrients. This was assessed via self-report, for example, food diaries. The cross-sectional nature of the studies meant that the direction of the relationship could not be determined. While it was generally assumed that family mealtimes predict eating behaviours, it is plausible that some eating behaviours (for example, a child’s willingness to try new food) influenced parents’ willingness to have family meals, and, therefore, their frequency.

Family meal frequency was most commonly investigated. Numerous reviews found that more frequent family meals were positively associated with improved nutrition [39]. On the other hand, the mixed findings in relation to family meal frequency found by others led them to conclude they were unrelated [11,23,40].

Beyond meal frequency, several reviews examined the influence of other family mealtime characteristics on nutrition. Sharing family meals was found to improve nutrition [3,8], as well as the presence of a family member [3,35], parental modelling [28], prioritisation of family meals [35] and a positive mealtime atmosphere [28,41]. Glanz et al.’s review [3] emphasised the benefits of eating family meals at home, rather than outside of the home, consistent with Berge et al.’s [35] finding that more frequent fast food consumption in family meals was associated with poorer nutrition.

Several reviews noted the role of TV during family meals, finding that the benefits to nutrition could be undone if the TV was on [2,8,10,12,24,25,28,32,35,42], although Berge et al. [35] argued that regular family meals with the TV on resulted in a healthier diet than irregular family meals.

#### 3.5.2. Family Meals as a Predictor of Weight-Status

Weight status was the most commonly assessed outcome, investigated by eleven systematic reviews. Original studies considered weight status because of its alignment with over-weight and obesity, although samples in most studies under-represented the proportion of overweight young people compared to current estimates [43,44]. Weight status was operationalised across original studies in a variety of ways, typically overweight, obesity, BMI or zBMI.

As with eating behaviours/nutrition, more frequent family meals were typically found to be inversely associated with obesity [2,3,8,9,22,35,40,45,46], although Dallacker et al. [27] noted that the impact was small. Reviews also found evidence for an association with other family mealtime characteristics; for example, obesity was less likely if more meals were eaten together [45], more meals were eaten at home [45], mealtime practices were more positive (for example, eating together without the TV on [45], and the mealtime environment was more positive [3,41]. Bates et al. [45] found obesity was less likely if mealtime routines were more predictable, although Beckers et al. [47] obtained inconsistent results, with a comparable number of studies finding non-significant associations between mealtime routine and with lower weight outcomes and one finding that mealtime regularity was associated with higher weight outcomes. Similarly, findings relating to the presence of parents were inconsistent, with Bates et al. [45] finding their presence protected against obesity while Dallacker et al. [27] did not.

Not all findings relating to family meals supported their positive role in health outcomes. Martin-Biggers et al. [2] found that a positive mealtime atmosphere was associated with increased energy intake, perhaps beyond what is required for energy balance, and Bates et al. [45] found that regular mealtimes were associated with greater likelihood of obesity.

### 3.6. Family Mealtimes as a Predictor of Psychosocial Outcomes

Seven systematic reviews examined the association between family meal frequency and psychosocial outcomes.

Glanz et al. [3] and Martin-Biggers [2] found a broadly positive association between family meals and risk behaviours. Goldfarb et al. [48] did not find consistent evidence for an association between family mealtime frequency and tobacco or alcohol use, although Harrison et al. [29] found that more frequent family meals were associated with less illicit drug use in females. Both Goldfarb et al. [48] and Harrison et al. [29] found that more frequent family meals were associated with less violence/delinquency.

Evidence for an association between more frequent family meals and better mental health/psychological well-being was relatively consistent, with reviews finding that more frequent family meals were associated with less depression [29,48], fewer body concerns [29], higher self-esteem [29], less disordered eating [2,3,10,29] and less suicidal ideation [29,48], although several original studies found these effects were moderated by adolescents’ sex. These findings reflect those of Skeer and Ballard [6], who found that a more negative emotional mealtime climate was associated with greater levels of disordered eating, and Dorol-Beauroy-Eustache [49] who found that more frequent family meals moderated the relationship between cyberbullying and internalising problems, including self-harm, suicide attempts and ideation. More frequent family meals were also found to be associated with adolescents’ improved perceptions of family relationships [2].

Evidence for an association between family meals and academic achievement was less clear than that for mental health outcomes. Burrows et al. [5] and Martin-Biggers et al. [2] did not find consistent evidence that frequent family meals were associated with academic achievement, while Glanz et al. [3] found that family meals were positively associated with a range of psychosocial outcomes, including academic achievement, and Harrison et al. [29] found that their frequency was associated with academic achievement, but only for females (not males). The samples, search criteria and conceptualisation of key constructs were broadly similar across these systematic reviews, so the reasons for differences in their findings are unclear.

### 3.7. Moderators of the Relationship between Family Mealtimes and Outcomes

Many reviews investigated socio-demographic characteristics that may moderate the relationship between family mealtimes and outcomes. Although two reviews found ethnicity to be a correlate of family meal frequency (see Section 3.4.1) [2,12], reviews investigating ethnicity as a moderator of the relationship between family meal frequency and weight/nutrition outcomes concluded that these findings were inconsistent [3,47] Findings were somewhat mixed in relation to the child/adolescent’s sex, with one review finding that more frequent family meals protected females (but not males) against disordered eating, substance use, body image concerns and suicidal thoughts [29], while others found that family meal frequency did not moderate their relationship with either nutrition or weight outcomes [27,28,47]. Findings relating to the role of socio-economic status as a moderator were also mixed [12,27,28], as were the characteristics of the family and parenting [3,12]. Four reviews limited their inclusion criteria to original studies conducted in the USA. Nearly all those remaining did not impose limitations relating to country but neither did they investigate country as a potential moderator. The one study that did found that country did not play a moderating role [26]. Reviews of original studies that considered several socio-demographic variables as moderators [3,33] typically found that any association between family meals and nutrition diminished once adjustments were made.

### 3.8. Interventions Aimed at Promoting Family Mealtimes

Four systematic reviews included original articles evaluating interventions aimed at promoting family meals [3,7,12,13]. In total, nine different interventions were evaluated. There was significant overlap between the reviews, with four original articles being included in more than one systematic review (Supplementary Table S4).

The nine evaluated interventions were programmes delivered to families and/or children/adolescents over a period of 1–10 months. Eight aimed to promote the frequency of family meals eaten at home, and one also targeted the healthiness of food served. The other targeted mealtime attitudes and communication. Eight were evaluated in randomised controlled trials or quasi-randomised controlled trials, while one relied on pre-post data.

Four of the evaluated interventions demonstrated a positive effect on family meal frequency, albeit small. One intervention did not impact family meal frequency but was associated with better dietary quality. One did not evaluate impact on family meal frequency but did demonstrate positive changes in mothers’ attitudes towards mealtimes and mother–child communication. The remaining three interventions failed to demonstrate an impact on family meals. The nine evaluated interventions used samples that varied somewhat in size and socio-demographic characteristics, and designs varied, with only some being randomised controlled trials; however, there do not appear to be consistent differences between those that were successful and those that were not.

### 3.9. Heterogeneity of Results

Many reviews concluded that findings across original studies were mixed; the conclusions of two were notably different [3,4]. However, it is of note that, where there were inconsistencies, they were almost exclusively between reviews that found an association and those that did not, rather than associations being found in opposite directions. There is a myriad of possible reasons for the differences across reviews but, given the widely commented-upon limitations relating to the measurement of constructs and the narrow range of potential confounders that is investigated, it is at least plausible that these inconsistencies are attributable to methodological differences.

## 4. Discussion

The aim of this umbrella review was to provide a comprehensive overview of the extensive literature on family mealtimes that integrated the research findings of different aspects. Our review questions are addressed as follows.

### 4.1. What Are the Characteristics of Family Mealtimes?

Family mealtimes have been characterised in a multitude of ways, both simply as a shared meal and with complexity in terms of where and with whom the meal is eaten. However, in many studies, family meals are not conceptualised or defined. Typically, family meals are operationalised in terms of frequency, most likely because a count of the number of family meals eaten per week is relatively straightforward to collect. There are variations across studies due to differences in the ways family meals are defined and operationalised, but it would seem that families typically eat together at least a few days per week, with only a small proportion not sharing meals at all. A consistent criticism made in the majority of systematic reviews relates to the lack of an encompassing definition of family meal and the lack of a reliable and valid measure.

### 4.2. What Are the Correlates of Family Mealtimes?

Numerous potential correlates have been investigated. More frequent family meals are associated with socio-demographic characteristics such as children being younger, families being dual-parent, higher socio-economic status, and possibly parents being more educated. Ethnicity may play a role, but it is difficult to draw general conclusions. The frequency of family meals is also influenced by their characteristics: family meals are eaten more frequently if they are shared with others and eaten in the kitchen/dining room without the TV on. Family environment plays a role in family meal frequency, particularly for adolescents. Meals are more frequent in families where parents adopt a more authoritative style of parenting, where eating together is prioritised, and where attitudes towards family meals are more positive. Similarly, family meals are more frequent when family functioning is better. Lack of time, due either to parents’ employment or family members’ busy lives, along with children’s disruptive behaviour, are both barriers to family meals.

### 4.3. What Outcomes Are Associated with Family Mealtimes?

A substantial proportion of research into family mealtimes has investigated their association with health and psychosocial outcomes. The evidence is consistent that more frequent family meals are associated with healthier eating behaviours and nutrition. Likewise, broader aspects of family meals are associated with healthier eating, for example, having meals with other family members or having home-prepared food. It is notable that several reviews found that this protective effect can be undone if the TV is on during family meals. Similarly, more frequent family meals have consistently been found to protect against obesity, although not all reviews support an entirely positive role for family meals as more frequent meals may also be associated with increased energy intake and obesity.

Findings relating family meal frequency to psychosocial outcomes are not entirely consistent but do suggest a protective effect in relation to adolescent risk behaviours including illicit drug use, violence and delinquency, psychological wellbeing, and academic achievement, although it likely that these associations are moderated by adolescents’ sex.

### 4.4. Are Interventions Aimed at Promoting Family Mealtimes Effective?

Despite the similarities between them, interventions aimed at promoting family mealtimes have had mixed results to date. It is unclear why some have been effective when others have not.

### 4.5. Strengths and Limitations of Systematic Reviews

Across all reviews, many findings were consistent. Where findings were inconsistent, it was generally that the results did not demonstrate an association rather than that they were contradictory.

The quality of reviews was variable; however, most adopted critical steps in systematic reviewing. Typically, review questions were clearly stated, the searches were systematic and comprehensive, and data were extracted in sufficient detail. A majority considered the risk of bias and heterogeneity of findings. Confidence in their findings could, therefore, be high.

Almost all systematic reviews noted that research was limited by the lack of a consistent definition of family meals, particularly one that encompassed mealtime characteristics beyond frequency and was sufficiently nuanced to capture changes. Equally commented upon was the lack of a standardised, valid and reliable measure that adequately captured family mealtimes. Most systematic reviews examining correlates of family mealtimes noted that studies were typically cross-sectional, meaning that direction and causality could not be determined, and only a narrow range of potential confounders were investigated. Indeed, Goldfarb et al. [48] concluded that much of the evidence indicating an association between family mealtimes and adolescents’ risky behaviours was an artifact of underlying confounders.

### 4.6. Strengths and Limitations of Umbrella Review

In terms of both methodology and reporting, this umbrella review followed best practices [18,19]. Nevertheless, although robustly designed and rigorously executed, it does have limitations. The search terms were designed to ensure all relevant systematic reviews that were published were retrieved, even if they did not describe themselves as a systematic review. Moreover, the systematic reviews included studies from across several countries and socio-demographic groups, ensuring a breadth of representation. However, articles were only included if they were published in English, and it is possible that relevant systematic reviews published in other languages were overlooked. We also used publication in peer-reviewed journals as a proxy for quality; indeed, the quality of the included reviews is a strength, but it is possible that some relevant, high-quality systematic reviews were missed. Finally, to avoid overlap, where systematic reviews included previous systematic reviews, findings were only considered once; however, it was not feasible to assess the overlap of original studies. Consequently, the extent to which systematic reviews included the same studies, effectively giving some findings undue weight, is unknown.

### 4.7. Implications for Practice, Policy and Research

Across the developed world, countries are struggling to halt rising levels of childhood obesity (for example, [50,51,52,53,54]). While government agency advice to families often references the benefit of shared meals (for example, [55]), with the exception of the European Union [56], few governments have explicitly included the promotion of family meals as part of their strategy to address childhood obesity (for example, [57,58]). This umbrella review reports clear evidence supporting a role for family meals in tackling children/adolescents’ overweight and obesity, as well as promoting their health and well-being more generally. Research should now build on this evidence and develop scalable interventions that support families to eat together. Although prioritisation and positive attitudes towards family meals are important, interventions should first aim to understand the barriers that families experience and seek to address them. It is especially important to understand the role of family functioning and mealtime environment to avoid the risk of increasing conflict in some families. Interventions that have already been demonstrated to be effective should be used as a starting point. For these interventions to be evaluated, it is essential that an encompassing definition of family meals is provided, and a valid and reliable assessment tool is developed.

## 5. Conclusions

By providing a comprehensive overview of research relating to family mealtimes, this umbrella review has established that more frequent family meals play a protective role in children and adolescents’ nutrition, weight status, risk behaviours, well-being, and academic achievement. Not only does integrating this extensive body of research ensure that researchers and practitioners are fully availed of the evidence, doing so enables easy identification of what is yet to be determined. The next steps will be to develop interventions promoting family meals that are scalable and provide the support that families say they need, along with a valid and reliable assessment tool so that their effectiveness can be evaluated.

## Figures and Tables

**Table 1:** Characteristics of included systematic reviews (*N* = 41).

Reference	Authors (Year of Publication)	Review Question(s) Pertinent to Family Mealtimes	Review Design	Key Findings Pertinent to Family Mealtime Research
[1]	McCullough et al., (2016)	How has the family meal environment been characterised in the existing literature?	Narrative 24 articles (33 unique datasets)	All studies reported ≥ 1 of the structural characteristics; most reported one, none reported all four. Frequency of family meals decreased between early childhood and adolescence (29 studies). Duration of family meals increased between early childhood and adolescence (4 studies). Eating at the table (location) decreased between early childhood and adolescence (3 studies). Presence of family members increased between early childhood and adolescence (7 studies). *Key limitation(s) pertinent to family mealtimes:* lack of consistent definitions and validated measures of family meals.
[2]	Martin-Biggers et al., (2014)	What methods have been used in family meal research? What are the links between family meals (frequency and atmosphere) and health, developmental, and BMI?	Narrative 81 articles	A range of definitions of family meals was used across studies, with some specifying the number of people who must be present and meal type (dinner). Parents experiences of family meals: Parents enjoyed and valued family meals but found them stressful and unpleasant, felt under pressure to provide them and found it difficult to make healthy meals. Barriers to frequency family meals included: employment, children’s after school activities, lack of meal planning, not having a regular time for meals, the inability of young children to sit still during meals, family members being hungry at different times, picky eaters. Parents overcame barriers to family meals by planning, making meals ahead of time, using time/effort saving appliances, creating the expectation family members will present, developing a structured mealtime. Parents with high cooking self-efficacy were more likely to overcome barriers relating to frequent family meals. Parents overcame resistance to family meals by making them enjoyable and minimally stressful, with some rewarding children for eating and insisting children eat specific foods. Correlates of family meals: Family meal frequency was positively associated with being of higher socio-economic status, having more education, being married, of Asian-American or Hispanic ethnicity, the presence of younger children in the family and an authoritative parenting style. Family meal frequency was negatively associated with parents’ employment, African-American ethnicity. Family meal frequency was positively associated with more traditional meal structure (meals eaten in the kitchen/dining room without the TV on) Family meal frequency as a predictor of outcome: There was limited evidence to support a decline in the frequency of family meals. More frequent family meals were associated with improved nutrition (increase in high-nutrient-dense food and decrease in low-nutrient-dense food) and unhealthy eating patterns (younger children). More frequent family meals were associated with less likelihood of overweight/obesity (interactions with sex and ethnicity). More frequent family meals protect against disturbed/disordered eating (adolescents) More frequent family meals were associated with improved perceptions of family relationships and fewer risk-taking behaviours (adolescents). Findings relating family meal frequency and children’s academic success were inconsistent. A positive mealtime atmosphere promoted greater energy intake. Watching TV during a family meal negated the benefits of frequent family meals. Interventions targeting family meals: Two studies evaluated interventions aimed at promoting family meals; both interventions were found to increase their frequency. *Key limitation(s) pertinent to family mealtimes:* inconsistencies in definitions of family meal and the lack of a valid measure to assess family meals.
[3]	Glanz et al., (2021)	What are the factors associated with in-home eating? What is the impact of in-home eating on the nutritional quality of meals? What is the relationship between in-home eating and child/adolescent outcomes? What is the influence of in-home eating on family relationships?	Narrative 65 studies (54 original studies (of which 4 articles assessed 4 interventions), 11 review articles)	Factors associated with more frequent in-home eating: Family structure (two-parent biological families with two or more children) Inconsistent findings relating to ethnicity, parent/child gender; child/adolescent’s age; socio-economic status; parents’ education. The impact of in-home eating on the nutritional quality of meals: Number/frequency of family meals was associated with diets of higher nutritional quality (possible interactions with age and ethnicity). Parental presence at family meals was associated with diets of higher nutritional quality. In-home meals were associated with diets of higher nutritional quality compared to meals eaten outside the home. The relationship between in-home eating and outcomes: Frequency of shared family meals was directly related to a reduced risk of childhood overweight/obesity, but the strength of the association was diminished when confounders or mediators (family, parenting and socio-demographic characteristics) are considered. Other aspects of in-home eating (mealtime characteristics, social interaction, mealtime environment) were positively associated with a reduced risk of childhood overweight/obesity. In-home eating was positively associated with other outcomes (academic, psychological, communication, risk behaviours, sleep, eating behaviour, restrained eating, eating disorders) although the relationship may be explained by family factors. In-home eating was associated with more positive emotions and less worry than meals eaten outside the home. The influence of in-home eating on family relationships: Findings linking in-home eating/shared meals and socio-demographic characteristics were mixed. Interventions aimed at promoting in-home eating: Four interventions programmes aimed at increasing in-home eating/shared meals demonstrated minimal to no effect. *Key limitation(s) pertinent to family mealtime research:* inconsistency in the measurement of shared meals at home.
[4]	Duriancik & Goff (2015)	Are children living in a single-parent household at risk of obesity?	Narrative 10 studies	Two studies investigated family meals. One investigated family meal frequency and found that children from single-mother homes had higher odds of infrequent family meals. The other investigated shared meals as a possible mechanism to explain the association between family structure and children’s higher risk of obesity but found it non-significant. *Key limitation(s) pertinent to family mealtimes:* Most studies were cross-sectional and causality could not be determined.
[5]	Burrows et al., (2017)	What are the effects of dietary intakes and behaviours on the academic achievement of school aged children?	Narrative 41 articles (40 studies)	One study reviewed assessed the frequency of family meals and found eating family meals ≥5 days per week was not associated with academic achievement. *Key limitation(s) pertinent to family mealtimes:* studies focused on household routines with few examining broader components of the home environment.
[6]	Skeer & Ballard (2013)	What mechanisms contribute to the protective effect against adolescent risk behaviour afforded by family meal frequency?	Narrative 18 articles	A majority of studies found an inverse relationship was found between family meal frequency and alcohol use for female adolescents. Findings from studies investigating the relationship between family meal frequency and tobacco and illicit drug use were mixed. Family meal frequency was likely to be inversely associated with adolescent aggressive/violent behaviour, school performance, sexual behaviour (moderated by ethnicity). More frequent family meals may be protective against mental health disorders among adolescents, and disordered eating patterns (female adolescents). *Key limitation(s) pertinent to family mealtimes:* studies were inconsistent in how family meal frequency was operationalised; only family meal frequency was assessed.
[7]	Dwyer et al., (2015)	What interventions (scope of strategies, settings, populations targeted) encourage more frequent family meals? What are the key correlates of and barriers to family meals?	Narrative 6 intervention articles (evaluating 6 interventions), 43 non-intervention articles	Six intervention studies evaluating multiple session programmes delivered to families (3) or children/adolescents (3). Of the 6, 4 demonstrated a positive effect on family meal frequency while 2 were not significantly related to family meals. Non-intervention articles showed: Family meal frequency was not related to the child’s sex (most studies). More frequent family meals were associated with more positive attitudes towards them. More frequent family meals were associated with the perception of them as more important (parents and adolescents) in all but one study. Findings relating to other sociodemographic characteristics were mixed (children’s age; parents’ age; ethnicity; rural vs. urban location; education; family structure; employment; psychosocial variables, home environment). Parents were aware of many benefits of family meals (increased communication, strengthened interpersonal relationships, the opportunity to model of healthy eating, provision of structure/routine) but described numerous barriers (time, scheduling, resource, accommodating preferences; adolescents’ desire for autonomy/dissatisfaction with family meals). *Key limitation(s) pertinent to family mealtimes:* none reported.
[8]	Fulkerson et al. (2014)	What are the associations between family meal frequency and children and adolescents’ dietary/weight outcomes?	Narrative 17 studies	Most family meals were prepared at-home. Family meals were associated with healthier food. Eating family meals while watching TV was associated with a less healthy diet. Family meal frequency was positively associated with diet quality. More frequent family meals were associated with less likelihood of higher BMI/overweight for some ethnic groups and/or sexes. *Key limitation(s) pertinent to family mealtimes:* definitions of family meal frequency were inconsistent across studies, and the impact of socio-economic status was largely investigated.
[9]	Tosatti et al., (2017)	Do family mealtimes have a protective effect on obesity and good eating habits in young people?	Narrative 15 articles	Across the included studies, a mean of one family mealtime was shared per day, with a wide range in prevalence of 27–81%. Most studies reported a favourable association between family mealtime frequency and BMI. Frequency of family mealtimes was associated with increased fruit, vegetable and diary intake, better regulation of appetite, healthier eating habits and a reduction in the consumption of fast food (adolescents). *Key limitation(s) pertinent to family mealtimes:* most studies focused only on the prevalence of family mealtimes.
[10]	Woodruff et al., (2008)	What are the patterns of family meals? How does the family meal influence adolescents’ dietary intake?	Narrative Almost 200 studies	Approximately from one-quarter to one-half of adolescents consumed family meals regularly (≥5 times per week), typically dinner. The number of family meals consumed per week declines with increasing age due to busy schedules (parents and adolescents), adolescents’ desire for autonomy, family relations, and not liking the food being served. Family meals are commonly consumed while watching TV, negatively impacting diet quality. Compared to parents and younger adolescents, older adolescents were less likely to eat family meals ≥ 5 per week and considered them less important. Family meal frequency was positively associated with diet quality, protects against increased body weight and disordered eating behaviours, and associated with a decreased likelihood of skipping breakfast. *Key limitation(s) pertinent to family mealtimes:* many potential mediating/confounding factors in the association between family meals and adolescent diet quality were not investigated, nor were aspects of family meals such as who prepared the meal, where the food was purchased, and who consumed the meal.
[11]	Robson et al., (2020)	What is the direction and magnitude of the exposure to family meals and: Child and adolescent dietary outcomes? Family functioning outcomes?	Family meal frequency: narrative 31 studies of which 8 were selected for meta-analysis Family functioning: narrative 12 studies of which 4 were selected for meta-analysis	Dietary Outcomes: Some evidence was found of a positive association between family meal frequency and intake of fruit, vegetables (when assessed separately or combined), sugar-sweetened beverages, and the diet quality. The association between family meal frequency and intake of snacks, fast food, and desserts was less clear. Family Functioning Outcomes: Evidence of a positive association between family meal/family dinner frequency and family functioning. *Key limitation(s) pertinent to family mealtimes:* lack of definitions and standardised measure for family meals.
[12]	Verhage et al., (2018)	Are characteristics of the family meal associated with outcomes in terms of health benefits in infants and toddlers?	Narrative 14 articles	Nine studies investigated the family meal frequency and parents’ perceptions of sharing meals: Most children had regular meals at the age of three.Mothers perceived mealtimes as a valuable moment to socialise.Family meals were more frequent among families that were Hispanic and non-Hispanic white compared to non-Hispanic black. In families with infants aged ≤12 months, family meals were more frequent when baby-leading was adopted compared to spoon-feeding. Not all meals were in the dining room, the TV was often on, less than half of mothers were present for the whole meal. Challenges included mess, child’s behaviour, difficulty planning a meal mothers’ physical/mental tiredness. Four studies examined associations between the family meal and children’s eating behaviour and diet quality: More frequent family meals were associated with more adaptive eating behaviours and better diet quality (interactions with the TV being on during the meal, socio-economic status, ethnicity and presence of mother during the meal). No studies examined the causal influences of family meals on child’s health. Interventions to promote family meals (2 studies): Watching a video about modelling healthy nutrition and communication during mealtimes resulted in positive changes in attitude towards family meals and improved maternal–child communication. Information resulted in an increase in family meals and a reduction in having the TV on during meals. *Key limitation(s) pertinent to family mealtimes:* lack of a standardised definition of family meal and variability in how family meals were operationalised.
[13]	Middleton et al., (2020)	What impact does the family meal have on the health and wellbeing of the family?	Narrative, meta-aggregation of qualitative studies 32 articles (17 qualitative articles, 15 intervention articles evaluating 9 interventions	Intervention Studies: Studies evaluated nine intervention programmes, ranging in duration from 1 to 10 months, targeted family meal frequency, environment and nutritional quality. Two interventions demonstrated statistically significant differences in family meal outcomes favouring intervention groups (meal frequency; amount of vegetables served, fruit and vegetable intake, added sugar intake). Qualitative Studies: There were a range of parental motivations for family meals including health, modelling and communication, and practical reasons. Families held different ideas about family meals in relation to what they are and the extent to which they should be prioritised. Parents used a range of strategies to bring family members together for a meal; for example, involving children, serving food that is liked. A range of factors influenced what is served including time, resources and schedules. Difficult/disruptive behaviour at mealtimes was common. Families used a range of strategies to persuade children to eat; for example, rules, pressure, reward and having the TV on. Families described a multitude of barriers to family meals including resource (including time and effort), scheduling and skills/confidence. *Key limitation(s) pertinent to family mealtimes:* lack of standardised measures of the family meal that adequately captured the changes, nuances and importance of the family meal.
[22]	Hammons & Fiese (2011)	What is the strength of the relationship between the frequency of shared family mealtimes and children’s nutritional health?	Meta-analysis 17 studies	Half of families shared meals 5-7 days per week; one third shared meals 1-4 days per week; 14% did not share any meals together. Children/adolescents in families who shared ≥3 meals per week are 12% less likely to be overweight than those who shared < 3 meals per week (not moderated by age). Older children in families who shared ≥3 meals per week have a 20% reduction in the odds of eating unhealthy foods compared to those who shared <3 meals per week (not true for younger children). Older children in families who shared ≥3 meals per week have a 24% increase in the odds of eating healthy foods compared to those who shared <3 meals per week (not true for younger children). Adolescents in families who shared ≥5 meals per week are 35% less likely to engage in disordered eating than those who shared <5 meals per week. Adolescents/children in families who had ≥5 meals per week were 25% less likely to have poor nutritional health (overweight/unhealthy eating/disordered eating) compared to those in families who had ≤1 meal per week (not moderated by age). Longitudinal studies suggest shared family mealtimes are associated with a 7% reduction in the likelihood of overweight and disordered eating at 5-year follow-up. Half of studies examining shared family meal frequency and overweight found no difference between ≥3 meals per week vs. <3 meals per week. Frequency of family meals favourably impacted diet quality. *Key limitation(s) pertinent to family mealtimes:* lack of precision in the measurement of the frequency and structural aspects of the family meal.
[23]	Pearson et al., (2009)	What are the correlates of the family environment associated with children’s and adolescent’s breakfast behaviour?	Narrative 24 articles (33 unique datasets)	One study examined frequency of family evening meals with a parent present and found a positive association with the likelihood of adolescents’ breakfast consumption. *Key limitation(s) pertinent to family mealtimes:* none reported.
[24]	Scaglioni et al., (2018)	How does the family environment influence children’s eating behaviours?	Narrative 88 studies	Frequency of family meals was linked with socio-demographic characteristics (not specified). Frequent family meals were favourably associated with food and nutrient intake, obesity, disturbed/disordered eating practices and consumption of healthy foods in all age groups and fewer risk-taking behaviours among adolescents. The TV on during family meals was associated with a reduction in the likelihood of fruit and vegetables being served. *Key limitation(s) pertinent to family mealtimes:* none reported.
[25]	van der Horst et al., (2017)	Which environmental correlates have been studied in relation to child and adolescent obesity-related behaviours? Which environmental factors are consistently associated with these obesity-related dietary behaviours?	Narrative 58 articles (77 unique datasets)	Three studies reported findings relating to family meals: One investigated family meal frequency and found that children from single-mother homes had higher odds of infrequent family meals. One study identified shared meals as a possible mechanism to explain the association between family structure and children’s higher risk of obesity but found it non-significant. One study found having the TV on during family meals was negatively associated with children’s intake of fruit, juice and vegetables, and positively associated with children’s intake of snacks, fast food and soft drinks. *Key limitation(s) pertinent to family mealtimes:* lack of longitudinal studies; measures lacked validity/objectivity.
[26]	Cislak et al., (2012)	What is the evidence for relationships between family variables, weight-related behaviours and body weight in children and adolescents?	Umbrella 18 systematic reviews	Of two reviews examining frequency of family meals (breakfast/dinner), both found that a higher frequency was positively associated with healthy diet in children/adolescents. *Key limitation(s) pertinent to family mealtimes:* none reported.
[27]	Dallacker et al., (2019)	What are the frequently investigated mealtime components in observational studies assessing the relationship between family meals and nutritional health? How strong is the relationship between the identified mealtime components and children’s nutritional health? Do characteristics such as age, outcome, and socio-economic status moderate the association between different mealtime components and children’s nutritional health?	Meta-analysis 50 studies	Six mealtime components were examined in most studies: TV on during family meals, parental modelling, food quality, positive mealtime atmosphere, children’s involvement in meal preparation, duration of family meals. All mealtime components were associated with better nutritional health (turning off the TV during family meals, parental modelling of healthy eating habits, higher diet quality and positive mealtime atmosphere), although effect sizes were small. Neither age (children vs. adolescents), outcome (BMI vs. diet quality), socio-economic status (controlled for vs. not controlled for) or study quality moderated the association between mealtime components and nutritional health. *Key limitation(s) pertinent to family mealtimes:* observational designs meant that the direction of the association between mealtime components and diet quality could not be determined and there was variability in the definition and operationalisation of family mealtimes.
[28]	Dallacker et al., (2018)	What are the nutritional health correlates of family meals? What is the impact of demographic and mealtime characteristics on the association between meal frequency and nutritional health?	Meta-analysis 57 studies	Frequent family meals were associated with lower BMI, a healthier diet, a less unhealthy diet and better overall diet quality. The effect size of family meal frequency on BMI was larger in studies that did not control for socio-economic status. Family members present during the meal and meal type (breakfast, lunch, dinner) did not moderate the association between family meal frequency and BMI. Country and age of child/adolescent/young adult did not moderate the association between family meal frequency and BMI. The impact of family meal frequency on BMI was small. Potential mechanisms for the association between family meal frequency and BMI: The number of family meals was negatively correlated with the number of ready-made dinners. Meals eaten alone/with friends were more likely to include fast food or ready-made food. Parental modelling or encouragement may influence children/adolescents/young people’s dietary behaviour. *Key limitation(s) pertinent to family mealtimes:* there was no agreed definition of family meal and limited use of a validated measure for family meal frequency.
[29]	Harrison et al., (2015)	What is the relationship between family meals and psychosocial outcomes in children and adolescents and are their differences between males and females?	Narrative 14 articles (9 unique datasets)	Family meal frequency rate ranged from 33 to 61%, with 11–33% of families having ≤2 meals per week. This is moderated by age (frequency of family meals decreases with age) and geographic location/culture (family meals are more common in Spain/Canada than in USA/UK). Studies that reported results by sex demonstrated more frequent family meals were associated with less disordered eating for females (not males). An inverse association was found between family meal frequency and substance use (females) and violence. An inverse association was found between family meal frequency and body image concern (females), self-esteem/self-efficacy, academic achievement, depressive symptoms, thoughts of suicide (females). *Key limitation(s) pertinent to family mealtimes:* most studies were cross-sectional so direction/causality could not be determined.
[30]	Rahill et al., (2020)	What is the role and responsibility of fathers in child feeding and what are the factor associated with paternal responsibility in child feeding? How does paternal modelling, diets and feeding practices relate to children’s eating behaviours and dietary intake? What are maternal perceptions of paternal feeding roles and how do maternal and paternal behaviours relate to children’s eating behaviour and dietary intake?	Narrative ~54 studies (number not reported)	Some roles relating to family mealtimes (cooking and grocery shopping) were shared between fathers and mothers. Fathers of young children believed that role modelling was important to promote healthy eating behaviours. Family mealtimes allowed fathers to enforce rules around food and encourage children to consume the meal. *Key limitation(s) pertinent to family mealtimes:* a narrow conceptualisation of the family meal construct.
[31]	Fraser et al., (2011)	In relation to children’s weight gain, overweight and obesity: What paternal parenting variables have been studied? What do these studies reveal about the influence of paternal parenting variables? What are the methodological limitations of current approaches to study paternal influences?	Narrative 10 studies	One qualitative study identified two themes relating to family mealtimes: mealtime rituals and routines; tension during mealtimes and found a variety of strategies are required to establish healthy eating patterns and consistent routines. *Key limitation(s) pertinent to family mealtimes:* small, unrepresentative samples.
[32]	Liu et al., (2009)	How does the family influence adolescent eating habits in terms of knowledge, attitudes and practices?	Meta-syntheses 48 studies	Family rules such as having vegetables with each meal, finishing everything on the place, and serving the same meal to all family members were found to facilitate adolescents’ eating habits. Regular meal schedules and eating with the family were proposed as strategies to facilitate healthy adolescents’ eating habits. The benefits of home meals could be counteracted by unstructured practices, namely accommodating taste preferences of individual family members and failing to negotiate for healthy eating. An unpleasant atmosphere arising from poor family relationships sometimes prevented adolescents from eating at home. *Key limitation(s) pertinent to family mealtimes:* none reported.
[33]	Valdés et al., (2013)	What is the relationship between the frequency of family meals and the risk of overweight in children and adolescents?	Narrative 15 articles	While several studies found an inverse relationship between frequency of family meals and BMI/overweight, the association became non-significant once adjustments were made for potentially confounding variables (age, gender, socio-economic status, diet, physical activity). *Key limitation(s) pertinent to family mealtimes:* lack of definition of family meal and scarce information about the characteristics of family meals.
[34]	Khandpur et al., (2014)	What are the methodological characteristics of studies assessing fathers’ feeding practices? What are the general patterns in fathers’ feeding practices and how do they differ from mothers? What are the child–parent correlates of fathers’ feeding practices?	Narrative 20 studies	Fathers reported lower perceived responsibility for child feeding compared to mothers. Fathers reported eating meals with children less frequently than mothers. *Key limitation(s) pertinent to family mealtimes:* most studies were cross-sectional so direction/causality could not be determined.
[35]	Berge et al., (2009)	What are the familial correlates of child and adolescent obesity?	Narrative 81 studies	Correlates of family meal frequency: Higher prioritisation of meal structure and social eating was associated with more frequent family meals in young adulthood. Greater family connectedness was associated with more frequent family meals. More frequent family meals were associated with a healthier diet: More fruit and vegetables, grains, calcium-rich foods, protein, fibre, macronutrients, micronutrients. Less fried food and soda, saturated and trans-fat, and lower glycaemic load. In addition to family meal frequency, prioritisation of family meals and the presence of a family member were associated with: Higher consumption of fruit, vegetables, diary (adolescents). Less likelihood of skipping breakfast (adolescents). Less likelihood of dieting, extreme weight-control behaviours and chronic dieting (girls and young adult females). More frequent family meals were associated with: Reduced odds of being overweight 1- and 3-years later. TV on during family meals was associated with a less healthy diet, unhealthy dietary outcomes and obesity (adolescents). <3 fast-food family meals per week was associated with a greater likelihood of having vegetables and milk with meals compared to ≥3 fast-food family meals per week. *Key limitation(s) pertinent to family mealtimes:* covariates assessed limited to gender, socio-economic status and ethnicity and possibly influential covariates were omitted; family meals were assessed using self-report and important characteristics of family meals (breakfast, lunch or dinner; parental presence; home-cooked vs. pre-packaged/fast-food) were not investigated.
[36]	Vollmer & Mobley (2013)	What is the relationship between parenting and/or feeding styles on child body weight and/or child obesogenic behaviours?	Narrative 51 studies	One of five studies examining the role of family meal characteristics found an authoritative parenting style was positively associated with family meal frequency. *Key limitation(s) pertinent to family mealtimes:* None reported.
[37]	Jenkins & Horner (2005)	What are the barriers that influence eating behaviours in adolescence?	Narrative 20 articles (11 studies, 6 reviews, 3 intervention studies)	Frequency of family meals favourably impacted diet quality. Mothers’ employment outside the home and parents’ work schedules resulted in decreased time for meal preparation and may influence meal planning and food preferences. *Key limitation(s) pertinent to family mealtimes:* intervention studies did not address parental influences/involvement in adolescent nutrition (e.g., shaping eating patterns, preparing meals).
[38]	Titis (2022)	What are parents’ perspectives of the impact of the COVID-19 lockdown on the family food environment and food-related activities?	Narrative 14 studies	Changes arising from the COVID-19 lockdown were associated with parents having more time, greater interest in health and nutrition, increased motivation to eat more healthily. Specifically: An increase in meal planning; An increase in home-cooked (associated with higher-income and dual-parent families); An increase in variety and complexity of meals; An increase in healthiness of meals; An increase in child involvement in meal preparation; Eating more meals with children; A more positive mealtime environment; An increase in eating in as opposed to eating out. *Key limitation(s) pertinent to family mealtimes:* quality of studies included not assessed.
[39]	Do Amaral e Melo et al., (2020)	What is the association between family meals frequency and food consumed and/or children’s, adolescents’ and young adults’ nutritional status?	Narrative 50 studies	Studies varied in the operationalisation of meal frequency, assessing the frequency of meals in general, dinner/supper/evening meal, dinner only, all three main meals (breakfast, lunch, and dinner), breakfast only, breakfast and dinner, lunch and dinner with most assessing meal frequency over a 1-week period. Association between family meal frequency and nutritional status: A majority of studies found an association between frequent family meals and lower incidence/prevalence of overweight (interactions with sex and ethnicity). A minority of studies found no association between frequency of family meals and nutritional status. Few studies found an association between frequent family meals and weight gain for some ethnicities/ages. Association between family meal frequency and food consumed/diet quality: Most studies found more frequent family meals were associated with increased consumption of healthy dietary practices and/or diet quality and decreased unhealthy dietary practices. Some studies found no association between family meal frequency and diet/dietary quality. *Key limitation(s) pertinent to family mealtimes:* studies did not consider other meal characteristics (e.g., duration, location, type of meal, food served).
[40]	Krølner et al., (2011)	What are children/adolescents’ views and experiences regarding determinants of their intake of fruit and vegetables?	Meta-syntheses 31 studies	Children/adolescents perceived that family dinner at home is the only appropriate time to eat vegetables. *Key limitation(s) pertinent to family mealtimes:* none reported.
[41]	Pearson et al., (2008)	What are the correlates of the family environment associated with children’s and adolescent’s fruit and vegetable intake?	Narrative 60 articles (88 unique datasets)	Findings relating adolescent vegetable consumption to the frequency of family meals in general, family breakfast and family dinner were inconsistent, with most studies finding they were unrelated. Fast-food bought for the family meal was investigated in one study and was found to be unrelated to vegetable consumption. *Key limitation(s) pertinent to family mealtimes:* most studies were cross-sectional so direction/causality could not be determined; many studies did not report the reliability/validity of measures used to assess sociocultural family correlates.
[42]	Rasmussen et al., (2006)	What are the determinants of fruit and vegetable consumption in children and adolescents?	Meta-analysis and narrative 98 articles	Five of six papers investigating the influence of shared family meals found a positive association with children’s consumption of fruit and/or vegetables. The sixth paper found no association. *Key limitation(s) pertinent to family mealtimes:* many studies were based on small, non-representative samples; the validity of instruments used to assess constructs was reported either superficially or not at all.
[43]	Smith et al., (2022)	What are the child/adolescent level correlates of mealtime emotional climate?	Narrative 14 studies	A more positive mealtime emotional climate was associated with higher consumption of healthy food. A more negative mealtime emotional climate was associated with the consumption of unhealthy food. A positive mealtime emotional climate was favourable associated with BMI/weight status. A more negative mealtime emotional climate was generally associated with greater levels of disordered eating (girls and adolescents). *Key limitation(s) pertinent to family mealtimes:* none reported.
[44]	Avery et al., (2017)	What are the associations between watching TV during a meal or while consuming a snack, and children’s diet quality?	Narrative 13 studies	Having the TV on during the family meal was associated with poorer diet quality in terms of adolescents’ consumption of fried foods (girls), soft drinks, grains, calcium-rich foods, and vegetables. *Key limitation(s) pertinent to family mealtimes:* none reported.
[45]	Bates et al., (2018)	What relations exist between the organisation of the family home environment and child obesity?	Narrative 32 studies	10/16 studies found a significant relation between meal routines and child weight, 70% of which was in the expected direction. Less likelihood of obesity if: Dinner eaten together. Parent present at the meal. More predictable dinnertime routines. More frequent family meals. Fewer meals outside the home. Greater likelihood of obesity if: Regular mealtimes. Fewer positive family meal practices (e.g., eating as a family at home without distractions such as TV or smart phones). Lower home breakfast frequency. *Key limitation(s) pertinent to family mealtimes:* studies focused on household routines with few examining broader components of the home environment.
[46]	Psaltopoulou et al., (2019)	What is the observational and/or interventional evidence for nutritional, physical activity and behavioural factors preventing and/or treating child and adolescent obesity?	Umbrella 66 articles	One meta-analysis investigated the frequency of family meals and childhood obesity and found that children having ≥3 family meals per week were 12% less likely to become obese compared to children who had <3 family meals per week. *Key limitation(s) pertinent to family mealtimes:* none reported.
[47]	Beckers et al., (2021)	What are the prospective links between food parenting practices and children’s weight outcomes?	Narrative 38 articles (28 unique datasets)	Nine studies (six independent datasets) examined the association between meal routines and weight outcomes. Some studies found ethnicity to be a moderator meal frequency and weight outcomes but, taken together, it is unclear which ethnicities might benefit from a higher meal frequency. Age was not found to be a moderator. Inconsistent findings and the quality of studies mean the prospective associations between aspects of meal routines and weight outcomes are unclear. *Key limitation(s) pertinent to family mealtimes:* often, studies did not adjust for important confounders and/or used non-validated instruments to measure food parenting practices.
[48]	Goldfarb et al., (2015)	What role does the family meal play in adolescent risk behaviours?	Narrative 14 articles	Most studies operationalised family meals as the frequency of either family dinners or family meals in general. Studies that dichotomised family dinner/meal frequency typically considered ≥5 meals per week as regular/frequent. Most studies controlled for basic demographic and socioeconomic characteristics; some studies controlled for family connectedness. No consistent association was found between frequent family meals and alcohol/tobacco use. Some studies found associations between frequent family meals and substance use (approximately half), violence/delinquency (approximately one third) and depression/suicide ideation (two of two studies) in some studies. Findings were influenced by adolescents’ sex and the use of empirical techniques that minimised the effect of bias from potential confounders. *Key limitation(s) pertinent to family mealtimes:* lack of homogeneity in how family meals were measured.
[49]	Dolor-Beauroy-Eustache & Mishara (2021)	What factors influence the impact of cyberbullying on suicidal and self-harm behaviours among children and adolescents?	Narrative 66 studies	A single study investigating the role of family meals showed that family dinners moderated the relationship between cyberbullying and internalising problems (including self-harm, suicide attempts and ideation). *Key limitation(s) pertinent to family mealtimes:* Most studies were cross-sectional so direction/causality could not be determined; studies used different instruments and conceptualisations to measure cyberbullying and suicidal and self-harm behaviours.

**Table 2:** Constructs assessed by included systematic reviews (*N* = 41).

Reference	Author (Year of Publication)	Constructs (n)	Characteristics of Investigated Family Mealtimes (11 Reviews)	Correlates of Investigated Family Mealtime Characteristics (18 Reviews)	Investigated Outcome(s) that Family Mealtimes Predict (33 Reviews)	Interventions Promoting Family Mealtimes (4 Reviews)
[1]	McCullough et al., (2016)	1		location; duration; presence of family members		
[2]	Martin-Biggers et al., (2014)	3	parents’ experiences	socio-demographics; meal structure, location, TV on; barriers/facilitators	BMI/weight status; psychosocial	
[3]	Glanz et al., (2021)	3		previous family meal frequency; ethnicity	BMI/weight status	4 interventions evaluated
[4]	Duriancik & Goff (2015)	2		single-mother homes	BMI/weight status	
[5]	Burrows et al., (2017)	1			psychosocial	
[6]	Skeer & Ballard (2013)	1			psychosocial	
[7]	Dwyer et al., (2015)	3	parents’ experiences	socio-demographics; barriers/facilitators		6 interventions evaluated
[8]	Fulkerson et al., (2014)	2	meal frequency; TV		nutrition; BMI/weight status	
[9]	Tosatti et al., (2017)	2	meal frequency		eating behaviour; nutrition; BMI/weight status	
[10]	Woodruff et al., (2008)	3	meal frequency; TV	socio-demographics	nutrition	
[11]	Robson et al., (2020)	2		family functioning	nutrition; psychosocial	
[12]	Verhage et al., (2018)	3	meal frequency; location; TV; presence of parent	ethnicity; feeding approach	eating behaviour; nutrition	2 interventions evaluated
[13]	Middleton et al., (2020)	3	meal frequency; parents’ motivations, perceptions, strategies	barriers/facilitators		9 interventions evaluated
[22]	Hammons & Fiese (2011)	2	meal frequency		nutrition	
[23]	Pearson et al., (2009)	1			nutrition	
[24]	Scaglioni et al., (2018)	2		socio-demographics	eating behaviour; nutrition; psychosocial	
[25]	van der Horst et al., (2017)	2		single-mother homes	eating behaviours; nutrition	
[26]	Cislak et al., (2012)	1			nutrition	
[27]	Dallacker et al., (2019)	1			nutrition; BMI/weight status	
[28]	Dallacker et al., (2018)	1			nutrition	
[29]	Harrison et al., (2015)	3	meal frequency	socio-demographics	psychosocial	
[42]	Rasmussen et al., (2006)	1			nutrition	
[30]	Rahill et al., (2020)	2	parents’ roles			
[31]	Fraser et al., (2011)	1	fathers’ experiences			
[32]	Liu et al., (2009)	2		atmosphere at home	eating behaviours	
[33]	Valdés et al., (2013)	1			nutrition; BMI/weight status	
[34]	Khandpur et al., (2014)	2		food parenting practices	BMI/weight status	
[35]	Berge et al., (2009)	2		prioritisation of family meals; family connectedness	nutrition; BMI/weight status	
[36]	Vollmer & Mobley (2013)	1		parenting style		
[37]	Jenkins & Horner (2005)	2		mothers’ employment	nutrition	
[38]	Titis (2022)	1		COVID-19 pandemic restrictions		
[39]	Do Amaral e Melo et al., (2020)	1			nutrition	
[40]	Krølner et al., (2011)	1			eating behaviours	
[41]	Pearson et al., (2008)	1			eating behaviours	
[43]	Smith et al., (2022)	1			eating behaviour; nutrition; BMI/weight status	
[44]	Avery et al., (2017)	1			nutrition	
[45]	Bates et al., (2018)	1			BMI/weight status	
[46]	Psaltopoulou et al., (2019)	1			BMI/weight status	
[47]	Beckers et al., (2021)	1			nutrition	
[48]	Goldfarb et al., (2015)	1			psychosocial	
[49]	Dolor-Beauroy-Eustache & Mishara (2021)	1			psychosocial	

## Data Availability

Not applicable.

## Supplementary Materials

The following supporting information can be downloaded at: https://www.mdpi.com/article/10.3390/nu15132841/s1, Table S1: excluded studies (n = 27); Table S2: key search criteria of included reviews (n = 41); Table S3: conceptualisation, definition and/or operationalisation of family mealtime and other key constructs of included reviews (n = 41); Table S4: extent to which systematic reviews were included in other reviews (n = 41); Table S5: quality of included reviews (AMSTAR-2) (n = 41).
