# Supplementary material for: Family Mealtimes: A Systematic Umbrella Review of Characteristics, Correlates, Outcomes and Interventions
Source: Nutrients. 2023 Jun 22;15(13):2841. doi: 10.3390/nu15132841 (PMC10346164; doi:10.3390/nu15132841)
Supplement: Supplementary file 1 [file nutrients-15-02841-s001.zip › Supplementary Table S1.pdf]

Supplementary Table S1. Studies Excluded (n = 27)

Citation	Reason
Balantekin, K. N., Anzman-Frasca, S., Francis, L. A., Ventura, A. K., Fisher, J. O., & Johnson, S. L. (2020). Positive parenting approaches and their association with child eating and weight: A narrative review from infancy to adolescence. *Pediatric obesity*, *15*(10), e12722.	1
Bennett, G., Young, E., Butler, I., & Coe, S. (2021). The impact of lockdown during the COVID-19 outbreak on dietary habits in various population groups: a scoping review. *Frontiers in nutrition*, *8*, 626432.	2
Fiese, B. H. (2012). Family mealtime conversations in context. *Journal of Nutrition Education and Behavior*, *44*(1), e1.	2
Golan, M., & Crow, S. (2004). Parents are key players in the prevention and treatment of weight-related problems. *Nutrition reviews*, *62*(1), 39-50.	2
Knobl, V., Dallacker, M., Hertwig, R., & Mata, J. (2022). Happy and healthy: How family mealtime routines relate to child nutritional health. *Appetite*, *171*, 105939.	2
Le Moal, F., Michaud, M., Hartwick-Pflaum, C. A., Middleton, G., Mallon, I., & Coveney, J. (2021). Beyond the normative family meal promotion: a narrative review of qualitative results about ordinary domestic commensality. *International Journal of Environmental Research and Public Health*, *18*(6), 3186.	2
Patrick, H., & Nicklas, T. A. (2005). A review of family and social determinants of children's eating patterns and diet quality. *Journal of the american college of nutrition*, *24*(2), 83-92.	2
Prangthip, P., Soe, Y. M., & Signar, J. F. (2019). Literature review: nutritional factors influencing academic achievement in school age children. *International journal of adolescent medicine and health*, *33*(2), 20180142.	2
Roblin, L. (2007). Childhood obesity: food, nutrient, and eating-habit trends and influences. *Applied Physiology, Nutrition, and Metabolism*, *32*(4), 635-645.	2
Verduci, E., Bronsky, J., Embleton, N., Gerasimidis, K., Indrio, F., Köglmeier, J., ... & ESPGHAN Committee on Nutrition. (2021). Role of dietary factors, food habits, and lifestyle in childhood obesity development: a position paper from the European Society for Paediatric Gastroenterology, Hepatology and Nutrition Committee on Nutrition. *Journal of Pediatric Gastroenterology and Nutrition*, *72*(5), 769-783.	2
White, A. A., Colby, S. E., Franzen-Castle, L., Kattelmann, K. K., Olfert, M. D., Gould, T. A., ... & Yerxa, K. (2019). The iCook 4-H study: an intervention and dissemination test of a youth/adult out-of-school program. *Journal of nutrition education and behavior*, *51*(3), S2-S20.	2
Larson, N., & Story, M. (2009). A review of environmental influences on food choices. *Annals of Behavioral Medicine*, *38*(suppl_1), s56-s73.	2
Langdon-Daly, J., & Serpell, L. (2017). Protective factors against disordered eating in family systems: a systematic review of research. *Journal of eating disorders*, *5*(1), 1-15.	3
Shirazi, Morvarid Ghasab, et al. "A review on determinants of nutritional behavior in teenagers." *Iranian Journal of Pediatrics* 27.3 (2017).	3
Perdew, M., Liu, S., & Naylor, P. J. (2021). Family-based nutrition interventions for obesity prevention among school-aged children: a systematic review. *Translational Behavioral Medicine*, *11*(3), 709-723.	3
Shepherd, J., Harden, A., Rees, R., Brunton, G., Garcia, J., Oliver, S., & Oakley, A. (2006). Young people and healthy eating: a systematic review of research on barriers and facilitators. *Health education research*, *21*(2), 239-257.	3
Zahedi, H., Djalalinia, S., Sadeghi, O., Zare Garizi, F., Asayesh, H., Payab, M., ... & Qorbani, M. (2022). Breakfast consumption and mental health: a systematic review and meta-analysis of observational studies. *Nutritional neuroscience*, *25*(6), 1250-1264.	3
Zeiher, J., Varnaccia, G., Jordan, S., & Lange, C. (2016). What are the determinants of childhood obesity? A literature review as part of the project "Nationwide monitoring of childhood obesity determinants". *Bundesgesundheitsblatt-Gesundheitsforschung-Gesundheitsschutz*, *59*, 1465-1475.	3
McClain, A. D., Chappuis, C., Nguyen-Rodriguez, S. T., Yaroch, A. L., & Spruijt-Metz, D. (2009). Psychosocial correlates of eating behavior in children and adolescents: a review. *International Journal of Behavioral Nutrition and Physical Activity*, *6*(1), 1-20.	3
Tabares-Tabares, M., Aznar, L. A. M., Aguilera-Cervantes, V. G., León-Landa, E., & López-Espinoza, A. (2022). Screen use during food consumption: Does it cause increased food intake? A systematic review. *Appetite*, 105928.	3
Thomas, J., Harden, A., Oakley, A., Oliver, S., Sutcliffe, K., Rees, R., ... & Kavanagh, J. (2004). Integrating qualitative research with trials in systematic reviews. *Bmj*, *328*(7446), 1010-1012.	3
Yee, A. Z., Lwin, M. O., & Ho, S. S. (2017). The influence of parental practices on child promotive and preventive food consumption behaviors: a systematic review and meta-analysis. *International Journal of Behavioral Nutrition and Physical Activity*, *14*(1), 1-14.	3
Pearce, A. L., Cevallos, M. C., Romano, O., Daoud, E., & Keller, K. L. (2022). Child meal microstructure and eating behaviors: A systematic review. *Appetite*, *168*, 105752.	3
Blaine, R. E., Kachurak, A., Davison, K. K., Klabunde, R., & Fisher, J. O. (2017). Food parenting and child snacking: a systematic review. *International Journal of Behavioral Nutrition and Physical Activity*, *14*(1), 1-23.	3
Gebremariam, M. K., Vaqué-Crusellas, C., Andersen, L. F., Stok, F. M., Stelmach-Mardas, M., Brug, J., & Lien, N. (2017). Measurement of availability and accessibility of food among youth: a systematic review of methodological studies. *International Journal of Behavioral Nutrition and Physical Activity*, *14*, 1-19.	3
Cruwys, T., Bevelander, K. E., & Hermans, R. C. (2015). Social modeling of eating: A review of when and why social influence affects food intake and choice. *Appetite*, *86*, 3-18.	3
González-Monroy, C., Gómez-Gómez, I., Olarte-Sánchez, C. M., & Motrico, E. (2021). Eating behaviour changes during the COVID-19 pandemic: A systematic review of longitudinal studies. *International journal of environmental research and public health*, *18*(21), 11130.	4

Reason: *1: not in English; 2: not a systematic review; 3 did not address a research question; 4: did not define meal*
