# Supplementary material for: Family Mealtimes: A Systematic Umbrella Review of Characteristics, Correlates, Outcomes and Interventions
Source: Nutrients. 2023 Jun 22;15(13):2841. doi: 10.3390/nu15132841 (PMC10346164; doi:10.3390/nu15132841)
Supplement: Supplementary file 1 [file nutrients-15-02841-s001.zip › Supplementary Table S2.pdf]

Supplementary Table S2. Key Search Criteria of Systematic Reviews Included (n = 41)

Reference	Authors (Year of Publication)	Key Search Criteria[1, 2]
1	McCullough *et al* (2016)	• To January 2016<br>• Participants aged 2-18 years:<br>▪ Early childhood (2-5 years)<br>▪ Middle/Late Childhood (6-11 years)<br>▪ Adolescence (12-18 years)<br>• Studies conducted in the USA only<br>• Designs excluded: reviews, qualitative
2	Martin-Biggers *et al* (2014)	• January 1999 to July 2013<br>• Participants children, youth<br>• Designs included: qualitative, quantitative
3	Glanz *et al* (2021)	• To May 2018<br>• Participants children, adolescents<br>• Designs included: original data or systematic review
4	Duriancik & Goff (2015)	• To January 2018<br>• Participants aged 0-18 years<br>• Designs included: original research
5	Burrows *et al* (2017)	• 1985 to April 2016<br>• Participants aged 5-18 years<br>• Designs included: observational, experimental<br>• Designs excluded: letters, thesis, dissertations, conference proceedings
6	Skeer & Ballard (2013)	• January 2000 to July 2012<br>• Participants aged 10-18 years<br>• Included participants with eating disorders<br>• Peer reviewed<br>• Studies conducted in USA<br>• Designs included: quantitative cohort, case-control, intervention, experimental
7	Dwyer *et al* (2015)	• January 2000 to May 2014<br>• Participants aged 5 years - adolescence<br>• Peer reviewed<br>• Designs included: original research<br>• Designs excluded: literature reviews
8	Fulkerson *et al* (2014)	• August 1992 – August 2012<br>• Participants children, adolescents<br>• Peer reviewed<br>• Designs included: studies, reviews
9	Tosatti *et al* (2017)	• 2000 to 2016<br>• Participants children and adolescents<br>• Included studies reported in English, Portuguese or Spanish<br>• Designs included: cross-sectional, cohort, systematic reviews
10	Woodruff *et al* (2008)	• Not reported<br>• Participants aged 13-19 years<br>• Designs included: original research articles
11	Robson *et al* (2020)	• To December 2018<br>• Participants aged 6-18 years<br>• Studies conducted in the USA only<br>• Designs excluded: qualitative, reviews, methodological studies, evaluations of interventions, non-analytic designs
12	Verhage *et al* (2018)	• Dates unspecified<br>• No language restriction<br>• Participants aged 0-3 years<br>• Peer reviewed<br>• Designs included: empirical data<br>• Designs excluded: reviews, case studies, sample size <10
13	Middleton *et al* (2020)	• 2008 to April 2020<br>• Participants aged 2-18 years<br>• High-income countries only

		• Designs included: experimental, quasi-experimental, qualitative<br>• Designs excluded: Cohort, cross-sectional, cross-sectional longitudinal, observational, pilot, and feasibility studies; systematic reviews, meta-analysis, umbrella reviews
22	Hammons & Fiese (2011)	• To 2009<br>• Participants children, adolescents<br>• Peer reviewed<br>• Study reports an odds ratio or data that could be transformed into and odds ratio<br>• Designs included: longitudinal, cross-sectional
23	Pearson *et al* (2009)	• To April 2008<br>• Participants 6-18 years<br>• Peer reviewed<br>• Designs excluded: intervention studies
24	Scaglioni *et al* (2018)	• 2011 to January 2018<br>• Participants aged 6 months-19 years<br>• Families with socio-economic advantage<br>• Industrialised countries<br>• Peer reviewed or edited book<br>• Designs included: primary studies or studies presenting secondary analysis of data
25	van der Horst *et al* (2017)	• January 1980 to December 2004<br>• Participants aged 3-18 years<br>• Study conducted in an established market economy<br>• Peer reviewed<br>• Designs excluded: intervention studies
26	Cislak *et al* (2012)	• January 1990-January 2010<br>• Participants aged 2-19 years<br>• Peer reviewed<br>• Designs included: systematic reviews, reviews, meta-analysis of quantitative studies<br>• Designs excluded: reviews focused solely on children 0-4 years, reviews of treatment programmes, dissertations, book chapters
27	Dallacker *et al* (2019)	• Not reported<br>• Participants children, adolescents, young adults<br>• English or German<br>• Published and unpublished studies<br>• ≥5 studies investigating the component<br>• Sufficient statistics reported to calculate an effect size<br>• Designs excluded: book chapter, review, qualitative
28	Dallacker *et al* (2018)	• To January 2017<br>• Participants children, adolescents, young adults<br>• English or German<br>• Published and unpublished studies<br>• Sufficient statistics reported to calculate an effect size<br>• Designs included: longitudinal, cross-sectional
29	Harrison *et al* (2015)	• To July 2011<br>• Participants children, adolescents<br>• Peer reviewed<br>• Designs included: cross-sectional, longitudinal, RCT<br>• Designs excluded: case studies, commentaries, narrative reviews
30	Rahill *et al* (2020)	• To December 2018<br>• Participants aged 0-18 years<br>• Peer-reviewed<br>• Designs included: quantitative, qualitative
31	Fraser *et al* (2011)	• To 2010<br>• Participants aged 0-12 years<br>• Designs included: quantitative, qualitative
32	Liu *et al* (2009)	• To July 2020<br>• Participants aged 10-19 years<br>• Designs included: qualitative studies<br>• Designs excluded: evaluation of activities, included results from quantitative methods, reviews, conference abstracts.

33	Valdés *et al* (2013)	• Since 2005<br>• Participants aged <18 years<br>• English or Spanish<br>• Peer reviews<br>• Designs included: original articles<br>• Designs excluded: qualitative, reviews, editorials
34	Khandpur *et al* (2014)	• To February 2014<br>• Participants aged 2-18 years<br>• Peer reviewed
35	Berge *et al* (2009)	• Since 2000<br>• Participants aged 0-18 years; age range for studies investigating family meals 5-12 years<br>• Designs included: empirical, quantitative<br>• observational, experimental<br>• Designs excluded: intervention study, case study, review, theoretical paper
36	Vollmer & Mobley (2013)	• To September 2012<br>• Participants aged ≤ 18 years<br>• Peer reviewed<br>• Designs included/excluded: not reported
37	Jenkins & Horner (2005)	• 1995-2004<br>• Participants adolescents; age range in of participants in original studies 2-19 years<br>• Designs included: exploratory studies, reviews<br>• Designs excluded: position statements, methodology studies
38	Titis (2022)	• To June 2022<br>• No language restriction<br>• Participants children and adolescents<br>• Peer reviewed and pre-prints<br>• Designs included: all study designs<br>• Designs excluded: literature, systematic or narrative reviews
39	Do Amaral e Melo *et al* (2020)	• To May 2020<br>• Participants older children, adolescents, young adults<br>• English, French or Spanish<br>• Designs included: quantitative<br>• Designs excluded: qualitative
40	Krølner *et al* (2011)	• To December 2010<br>• Participants aged 6-18 years<br>• Peer reviewed<br>• Designs included: qualitative<br>• Designs excluded: quantitative, reviews, unpublished manuscripts
41	Pearson *et al* (2008)	• To April 2008<br>• Participants 6-18 years<br>• Peer reviewed<br>• Designs excluded: intervention studies
42	Rasmussen *et al* (2006)	• 1966 to December 2005<br>• Participants aged 2-18 years<br>• Peer reviewed<br>• Designs included: cross-sectional, longitudinal cohort, randomised<br>• Designs excluded: case studies, commentaries, methods or questionnaire development, narrative/systematic reviews
43	Smith *et al* (2022)	• January 1980-April 2020<br>• Participants aged 0-18 years<br>• Peer reviewed<br>• Sample size for case-control studies ≥10 per group or for cohort studies ≥20<br>• Designs included: quantitative cohort, case-control, intervention, experimental
44	Avery *et al* (2017)	• January 2000 to July 2014<br>• Participants aged ≤ 18 years<br>• Designs excluded: reviews, intervention studies, longitudinal studies where other variables may have influenced food/drink consumption

45	Bates *et al* (2018)	• October 2016 to January 2017<br>• Participants aged 2-12 years<br>• Peer-reviewed<br>• Designs included: original data<br>• Designs excluded: reviews
46	Psaltopoulou *et al* (2019)	• To September 2015<br>• Participants children and adolescents<br>• Designs included: meta-analysis synthesising observational studies<br>• Designs excluded: meta-analysis synthesising interventional studies as well as observational studies
47	Beckers *et al* (2021)	• 1990 to October 2019<br>• Participants aged 2-18 years<br>• Peer-reviewed<br>• Designs included: prospective, quantitative<br>• Designs excluded: experimental, case-control, reviews
48	Goldfarb *et al* (2015)	• January 1990-September 2013<br>• Participants aged 11-18 years<br>• Studies conducted in the USA only<br>• Designs excluded: non-empirical
49	Dolor-Beauroy-Eustache & Mishara (2021)	• Since 1990<br>• Participants aged 9-18 years<br>• English, French or Spanish<br>• Peer reviewed<br>• Included clinical populations<br>• Designs excluded: book chapters, theses, dissertations, systematic reviews, commentaries, case studies, editorials, letters, research protocols, unpublished data

[1] Unless stated otherwise, restricted to English Language

[2] Unless stated otherwise, excluded participants with eating/feeding disorders and/or clinical populations
