# Supplementary material for: Family Mealtimes: A Systematic Umbrella Review of Characteristics, Correlates, Outcomes and Interventions
Source: Nutrients. 2023 Jun 22;15(13):2841. doi: 10.3390/nu15132841 (PMC10346164; doi:10.3390/nu15132841)
Supplement: Supplementary file 1 [file nutrients-15-02841-s001.zip › Supplementary Table S3.pdf]

Supplementary Table S3. Conceptualisation, Definition and/or Operationalisation of Family Mealtime and Other Key Constructs by Systematic Reviews Included (n = 41)

Reference	Authors (Year of Publication)	Review Question(s) Pertinent to Family Mealtimes	Conceptualisation/Definition/Operationalisation of Family Mealtime and Other Key Constructs Assessed[1]
1	McCullough *et al* (2016)	How has the family meal environment been characterised in the existing literature?	*Family meal* structural characteristics operationalised as:<br>1. Frequency<br>2. Duration<br>3. Location<br>4. Presence of family members
2	Martin-Biggers *et al* (2014)	1. What methods have been used in family meal research?<br>2. What are the links between family meals (frequency and atmosphere) and health, developmental, and BMI?	*Family meal* defined as those occasions when food is eaten simultaneously in the same location by more than one family member.<br><br>*Health, developmental and BMI outcomes* operationalised by studies reviewed as nutrient intake, disturbed/disordered eating practices family relationships, academic success, risk-taking behaviours obesity, BMI, overweight, weight status.
3	Glanz *et al* (2021)	1. What are the factors associated with in-home eating?<br>2. What is the impact of in-home eating on the nutritional quality of meals?<br>3. What is the relationship between in-home eating and child/adolescent outcomes?<br>4. What is the influence of in-home eating on family relationships?	*Family meals* were conceptualised as:<br>In-home eating, operationalised as meals eaten at home (without mention of shared or family), shared meals at home, family meals.<br><br>*Nutritional quality (diet quality)* operationalised as consumption of specific food groups e.g. fruit, vegetables, fibre.<br><br>*Child/adolescent outcomes* operationalised in the studies reviewed as health outcomes, psychosocial outcome.<br><br>*Family relationships operationalised* in the studies reviewed family functioning, parent-child communication.
4	Duriancik & Goff (2015)	Are children living in a single-parent household at risk of obesity?	*Family meal* operationalised infrequent family meals.<br><br>*Obesity* operationalised as weight, overweight or BMI/zBMI.
5	Burrows *et al* (2017)	What are the effects of dietary intakes and behaviours on the academic achievement of school aged children?	Conceptualisation/definition/operationalisation of *family meal* not reported.<br><br>*Academic achievement* operationalised in the studies reviewed as the extent to which students had reached educational targets.<br><br>*Dietary intake* operationalised in the studies reviewed as intake of specific food groups, diet quality, macro- or micro-nutrient intake.<br><br>*Dietary intake behaviours* operationalised as any meal consumption behaviour.
6	Skeer & Ballard (2013)	What mechanisms contribute to the protective effect against adolescent risk behaviour afforded by family meal frequency?	*Family meals* operationalised as frequency of family meals (including dinners).<br><br>*Adolescent risk behaviour* operationalised by studies reviewed as alcohol, tobacco, marijuana and other drugs, aggressive and/or violent behaviours, poor school performance, sexual behaviour, mental health problems, disordered eating patterns.
7	Dwyer *et al* (2015)	What interventions (scope of strategies, settings, populations targeted) encourage more frequent family meals?<br>What are the key correlates of and barriers to family meals?	*Family meal* conceptualised as shared meals between parents/caregivers and children.
8	Fulkerson *et al* (2014)	What are the associations between family meal frequency and children and adolescents' dietary/weight outcomes?	*Family Meals* defined as meals eaten with the family.<br><br>*Dietary/weight outcomes* operationalised in the studies reviewed as: fruit and vegetable intake, micronutrient intake, unhealthy food intake, dietary quality, food choices/frequency, fast-food restaurant use, BMI, BMI percentiles, weight, mortality.
9	Tosatti *et al* (2017)	Do family mealtimes have a protective effect on obesity and good eating habits in young people?	*Family meals* conceptualised as family mealtimes and defined as the act of eating simultaneously with family members. Most studies reviewed operationalised family mealtimes as<br><br>*Good eating habits* operationalised by studies reviewed as greater intake of fruit and vegetables, protein, calcium and lower intake of sweets, sugar sweetened beverages, family unition, self-regulation of appetite.<br><br>*Obesity* operationalised by studies reviewed as overweight, BMI.
10	Woodruff *et al* (2008)	What are the patterns of family meals and how does the family meal influence adolescents' dietary intake?	*Family meals* defined as food eaten together with other family members, usually with one adult present.<br><br>*Dietary intake* operationalised by studies reviewed as intake of fruit and vegetable, energy, dairy foods, soft drinks, fibre, micronutrients, fried food, fat, body weight, disordered eating, skipping breakfast.
11	Robson *et al* (2020)	What is the direction and magnitude of the exposure to family meals and:<br>1. child and adolescent dietary outcomes?<br>2. family functioning outcomes?	*Family meals* defined as a minimum of a child eating a meal with at least one other individual at home.<br>*Family meal* operationalised in terms of frequency/regularity and varied across studies reviewed from between ≥ 3 to ≥ 5 meals per week, general or by meal type (breakfast, lunch, or dinner).<br><br>*Dietary outcomes* operationalised as the frequency of consumption specific food (e.g. fruit, vegetables), diet quality, intake sugar sweetened beverages, snack foods, fast foods and desserts.<br><br>*Family functioning outcomes* operationalised in the studies reviewed as family connectedness/cohesion, communication, expressiveness, conflict, problem solving.
12	Verhage *et al* (2018)	Are characteristics of the family meal associated with outcomes in terms of health benefits in infants and toddlers?	*Family meal* defined as a social moment in the day during which food is eaten together with at least one family member.<br><br>*Health benefits* operationalised by studies reviewed as healthier food intake, better nutrition, family relationships, fewer eating problems.
13	Middleton *et al* (2020)	What impact does the family meal have on the health and wellbeing of the family?	*Family meal* defined as an occasion at set-times of day when most, if not all members of the immediate family eat food together.<br><br>*Health and wellbeing* operationalised as dietary quality, weight status, BMI, physical or psychological health markers.
22	Hammons & Fiese (2011)	What is the strength of the relationship between the frequency of shared family mealtimes and children's nutritional health?	Most studies reviewed operationalised *family meals* as the frequency meals were shared with other family members; a few operationalised *family meals* as the frequency of regular family meals with no reference to family members' presence.<br><br>*Nutritional health*:<br>• Overweight: defined as BMI at the ≥85th percentile.<br>• Food consumption: operationalised in the studies reviewed as the frequency healthy food (fruit and vegetables, multigrain, breakfast consumption) and of unhealthy food (soda, fast food, fried foods, sweets/candy, skipping breakfast, eating less than 2 fruits/vegetables per day) consumption.<br>• Disordered eating: operationalised in the studies reviewed as bingeing, purging, extreme and less extreme weight-control behaviours.
23	Pearson *et al* (2009)	What are the correlates of the family environment associated with children's and adolescent's breakfast behaviour?	*Family meal* conceptualised as a sociocultural correlate, operationalised by studies reviewed as frequency of family evening meals with parent present.<br><br>*Breakfast behaviour*, a proxy for diet quality, nutritional adequacy, health and psychosocial outcomes, operationalised as frequency of breakfast consumption and breakfast skipping combined.
24	Scaglioni *et al* (2018)	How does the family environment influence children's eating behaviours?	*Family meals* operationalised as frequency of family meals.

			Family environment conceptualised as parenting behaviours, operationalised as specific and general parenting behaviours.<br>*Child eating behaviours* defined as dietary intake, diet patterns, intakes of specific foods or beverages, food choices, food preferences, eating styles, eating behaviours.
25	van der Horst *et al* (2017)	1. Which environmental correlates have been studied in relation to child and adolescent obesity-related behaviours?<br>2. Which environmental factors are consistently associated with these obesity-related dietary behaviours?	*Family meals* operationalised as frequency of family meals.<br><br>*Environment* was defined as anything outside the individual.<br><br>*Obesity-related behaviours* operationalised as energy, fat (total and energy percent), fruit, vegetable, snack, fast food and soft drink intake.
26	Cislak *et al* (2012)	What is the evidence for relationships between family variables, weight-related behaviours and body weight in children and adolescents?	Conceptualisation/definition/operationalisation of *family meal* not reported.<br><br>*Weight-related behaviours* operationalised in the studies reviewed as physical exercise, sedentary behaviour, diet/nutrition, energy intake, intake of healthy food (e.g. fruit, vegetables), intake of unhealthy food (e.g. snacks, soda, fast food).<br><br>*Body weight* operationalised as weight, body mass index, obesity, overweight.
27	Dallacker *et al* (2019)	1. What are the frequently investigated mealtime components in observational studies assessing the relationship between family meals and nutritional health?<br>2. How strong is the relationship between the identified mealtime components and children's nutritional health?<br>3. Do characteristics such as age, outcome, and socio-9economic status moderate the association between different mealtime components and children's nutritional health?	Components of the *family meal* were defined as its social, environmental and behaviour attributes.<br><br>*Nutritional health* operationalised in the studies reviewed as BMI, healthy diet (portions of fruit and vegetables each day), unhealthy diet (consumption of sugar-sweetened beverages, fast food, unhealthy sweet/salty snacks), overall diet quality (assessed using Healthy Eating Index).
28	Dallacker *et al* (2018)	1. What are the nutritional health correlates of family meals?<br>2. What is the impact of demographic and mealtime characteristics on the association between meal frequency and nutritional health?	*Family meal* operationalised as meal frequency/regularity.<br><br>*Nutritional health* operationalised in the studies reviewed as BMI, healthy diet (portions of fruit and vegetables each day), unhealthy diet (consumption of sugar-sweetened beverages, fast food, unhealthy sweet/salty snacks), overall diet quality (assessed using Healthy Eating Index).
29	Harrison *et al* (2015)	What is the relationship between family meals and psychosocial outcomes in children and adolescents and are their differences between males and females?	Studies reviewed operationalised *family meals* as family meal frequency.<br><br>Psychosocial outcomes operationalised by studies reviewed as disordered eating behaviour (including extreme and less extreme weight control behaviour), externalising behaviours (substance use, violence), internalising behaviours (body image concern, self-esteem/self-efficacy, academic achievement, depressive symptoms/thoughts of suicide).
30	Rahill *et al* (2020)	1. What is the role and responsibility of fathers in child feeding and what are the factor associated with paternal responsibility in child feeding?<br>2. How does paternal modelling, diets and feeding practices relate to children's eating behaviours and dietary intake?<br>3. What are maternal perceptions of paternal feeding roles and how do maternal and paternal behaviours relate to children's eating behaviour and dietary intake?	Conceptualisation/definition/operationalisation of *family meal* not reported.<br><br>*Mealtime routines and rituals* defined in only one study as activities that encompass and govern the selection, preparation, and eating of food.<br><br>*Eating behaviour/dietary* intake operationalised in a variety of ways in the studies reviewed e.g. sugar-sweetened beverage consumption, fast-food consumption.
31	Fraser *et al* (2011)	In relation to children's weight gain, overweight and obesity:<br>1. What paternal parenting variables have been studied?<br>2. What do these studies reveal about the influence of paternal parenting variables?<br>3. What are the methodological limitations of current approaches to study paternal influences?	Conceptualisation/definition/operationalisation of *family meal* not reported.<br><br>*Weight gain, overweight and obesity* operationalised as weight, BMI.
32	Liu *et al* (2009)	How does the family influence adolescent eating habits in terms of knowledge, attitudes and practices?	*Family meals* conceptualised as a parenting style within the adolescent practice domain, operationalised as home meals.<br><br>Conceptualisation/definition/operationalisation of *eating habits* not reported.
33	Valdés *et al* (2013)	What is the relationship between the frequency of family meals and the risk of overweight in children and adolescents?	*Family meals* operationalised as frequency of family meals.<br><br>*Childhood/adolescent overweight* operationalised by studies reviewed as BMI, overweight, waist circumference, abdominal obesity, adiposity.
34	Khandpur *et al* (2014)	1. What are the methodological characteristics of studies assessing fathers' feeding practices?<br>2. What are the general patterns in fathers' feeding practices and how do they differ from mothers?<br>3. What are the child-parent correlates of fathers' feeding practices?	Conceptualisation/definition/operationalisation of *family meal* not reported.<br><br>*Feeding practices* conceptualised as responsive/child centred or unresponsive/parent-centred.
35	Berge *et al* (2009)	What are the familial correlates of child and adolescent obesity?	*Family meals* conceptualised as forming part of the family functioning domain, along with emotional closeness/connectedness and family weight teasing. Other domains include the parental domain and the sibling domain.<br><br>*Family meals* operationalised in most studies reviewed as meal frequency, in two studies as priority of family meals, and in one study as family presence at an evening meal.<br><br>*Familial correlates* operationalised as parenting style, parenting practices, parental control over the feeding environment, parental pressure/restricting of the feeding environment, parenting encouraging and modelling of health behaviours, family meals, family emotional closeness/connection, family weight teasing, sibling weight teasing.<br><br>*Obesity* operationalised as BMI, weight status, dietary intake, physical activity.
36	Vollmer & Mobley (2013)	What is the relationship between parenting and/or feeding styles on child body weight and/or child obesogenic behaviours?	Conceptualisation/definition/operationalisation of *family meal* not reported.<br><br>*Parenting styles* defined as the specific parenting behaviours and practices that influence child development.<br><br>*Feeding styles* defined as the specific parenting behaviours and practices related to child feeding.
37	Jenkins & Horner (2005)	What are the barriers that influence eating behaviours in adolescence?	Studies reviewed operationalised *family meals* as frequency of eating dinner with the family.<br><br>*Eating behaviours* conceptualised as nutritional intake and eating patterns.
38	Titis (2022)	What are parents' perspectives of the impact of the COVID-19 lockdown on the family food environment and food-related activities?	*Family meals* conceptualised as food environment/food related activities such as meal planning, meal preparation, eating together.
39	Do Amaral e Melo *et al* (2020)	What is the association between family meals frequency and food consumed and/or children's, adolescents' and young adult' nutritional status?	*Family meal* operationalised as main meal (breakfast, lunch, dinner/supper/evening meal).<br><br>*Food consumed* operationalised as the intake of nutrients and diet quality.<br><br>*Nutritional status* operationalised via anthropometric measurements.
40	Krølner *et al* (2011)	What are children/adolescents' views and experiences regarding determinants of their intake of fruit and vegetables?	Conceptualisation/definition/operationalisation of *family meal* not reported.<br><br>Conceptualisation/definition/operationalisation of *fruit and vegetable intake* not reported.

41	Pearson *et al* (2008)	What are the correlates of the family environment associated with children's and adolescent's fruit and vegetable intake?	*Family meal* conceptualised as a sociocultural correlate, operationalised by studies reviewed as frequency of family meal, frequency of family breakfast, frequency of family dinner, fast-food bought for family meal, and number of evening meals with parent present.<br><br>*Fruit and vegetable intake* operationalised as fruit, fruit juice and vegetable consumption.
42	Rasmussen *et al* (2006)	What are the determinants of fruit and vegetable consumption in children and adolescents?	*Family meals* conceptualised as shared family meals.
43	Smith *et al* (2022)	What are the child/adolescent level correlates of mealtime emotional climate?	Conceptualisation/definition/operationalisation of *family meal* not reported.<br><br>*Mealtime emotional climate* defined as the level of positive and negative interpersonal interactions and emotional expression during mealtimes.
44	Avery *et al* (2017)	What are the associations between watching TV during a meal or while consuming a snack, and children's diet quality?	Conceptualisation/definition/operationalisation of *family meal* not reported.<br><br>*Children's diet quality* operationalised by the studies reviewed as fruit and vegetable intake, fat, added sugar, sugar-sweetened beverages, caffeine, carbohydrates and grains, protein, vitamins and minerals, fried food, snacks, measures of diet quality.
45	Bates *et al* (2018)	What relations exist between the organisation of the family home environment and child obesity?	*Family meals* conceptualised as meal routines, operationalised by the studies reviewed using items from Child Eating Habit Questionnaire, Feeding Practices and Structure Questionnaire, Family Ritual Questionnaire, Positive Family Meal Practices, Family Food Behaviour Survey.<br><br>*Family home environment* operationalised as family organisation, family disorganisation, chaos, crowding, routines.<br><br>*Obesity* operationalised as overweight/obesity/BMI, zBMI.
46	Psaltopoulou *et al* (2019)	What is the observational and/or interventional evidence for nutritional, physical activity and behavioural factors preventing and/or treating child and adolescent obesity?	Conceptualisation/definition/operationalisation of *family meal* not reported.<br><br>*Obesity* operationalised by studies reviewed as weight change, BMI, zBMI, overweight/obesity status, fat mass reduction.
47	Beckers *et al* (2021)	What are the prospective links between food parenting practices and children's weight outcomes?	*Family meal* conceptualised as meal routines, operationalised by the studies reviewed as frequency/regularity.<br><br>*Food parenting practices* conceptualised as coercive control, structure and autonomy support.<br><br>*Weight outcomes* operationalised in the studies reviewed as (changes in BMI/zBMI/BMI percentiles, weight for length z-score (zWFL) weight status, weight trajectory, total fat mass, fat mass index, percentage body fat, waist circumference, waist-to-height ratio, triceps skinfold, subscapular skinfold.
48	Goldfarb *et al* (2015)	What role does the family meal play in adolescent risk behaviours?	*Family meals* operationalised as breakfast, lunch or dinner.<br><br>*Risk behaviours* operationalised as sexual activity, violence and delinquency, school performance, depression and suicide ideation, general risky behaviours, well-being.
49	Dolor-Beauroy-Eustache & Mishara (2021)	What factors influence the impact of cyberbullying on suicidal and self-harm behaviours among children and adolescents?	*Family meal* operationalised frequency of family dinners.<br><br>*Suicidal/self-harm behaviours* operationalised by studies reviewed as suicide attempts, suicidal ideation, suicide planning, measures of suicidality/suicide risk, self-harm, self-injury, deliberate self-harm, non-suicidal self-harm, self-cutting, self-burning.

[1] Body Mass Index (BMI), adjusted Body Mass Index (zBMI)
