# Supplementary material for: Family Mealtimes: A Systematic Umbrella Review of Characteristics, Correlates, Outcomes and Interventions
Source: Nutrients. 2023 Jun 22;15(13):2841. doi: 10.3390/nu15132841 (PMC10346164; doi:10.3390/nu15132841)
Supplement: Supplementary file 1 [file nutrients-15-02841-s001.zip › Supplementary Table S4.pdf]

Supplementary Table S4: Extent Systematic Reviews Were Included in Other Systematic Reviews (n = 41)

Reference	Authors (Year of Publication)	Earlier Review(s) Included:	Comment
1	McCullough *et al* (2016)	None	
2	Martin-Biggers *et al* (2014)	None	Retain
3	Glanz *et al* (2021)	Hammons & Fiese (2011); Martin-Biggers *et al* (2014); Skeer & Ballard (2013)	Some original findings more nuanced
4	Duriancik & Goff (2015)	None	
5	Burrows *et al* (2017)	None	
6	Skeer & Ballard (2013)	None	Superseded by Glanz *et al* (2021)
7	Dwyer *et al* (2015)	None	
8	Fulkerson *et al* (2014)	Hammons & Fiese (2011)	Some original findings more nuanced
9	Tosatti *et al* (2017)	None	
10	Woodruff *et al* (2008)	None	
11	Robson *et al* (2020)	None	
12	Verhage *et al* (2018)	None	Retain
13	Middleton *et al* (2020)	None	
22	Hammons & Fiese (2011)	None	Retain
23	Pearson *et al* (2009)	None	Retain
24	Scaglioni *et al* (2018)	None	Retain
25	van der Horst *et al* (2017)	None	
26	Cislak *et al* (2012)	van der Horst *et al* (2007); Pearson *et al* (2009a)	Original findings more nuanced
27	Dallacker *et al* (2019)	None	
28	Dallacker *et al* (2018)	None	
29	Harrison *et al* (2015)	None	
30	Rahill *et al* (2020)	None	
31	Fraser *et al* (2011)	None	
32	Liu *et al* (2009)	None	
33	Valdés *et al* (2013)	None	
34	Khandpur *et al* (2014)	None	
35	Berge *et al* (2009)	None	
36	Vollmer & Mobley (2013)	None	
37	Jenkins & Horner (2005)	None	
38	Titis (2022)	None	
39	Do Amaral e Melo *et al* (2020)	None	
40	Krølner *et al* (2011)	None	
41	Pearson *et al* (2008)	None	
42	Rasmussen *et al* (2006)	None	
43	Smith *et al* (2022)	None	
44	Avery *et al* (2017)	None	
45	Bates *et al* (2018)	None	
46	Psaltopoulou *et al* (2019)	None	
47	Beckers *et al* (2021)	None	
48	Goldfarb *et al* (2015)	None	
49	Dolor-Beauroy-Eustache & Mishara (2021)	None	
