# Supplementary material for: Family Mealtimes: A Systematic Umbrella Review of Characteristics, Correlates, Outcomes and Interventions
Source: Nutrients. 2023 Jun 22;15(13):2841. doi: 10.3390/nu15132841 (PMC10346164; doi:10.3390/nu15132841)
Supplement: Supplementary file 1 [file nutrients-15-02841-s001.zip › Supplementary Table S5.pdf]

Supplementary Table S5. Quality of Systematic Reviews Included (AMSTAR-2) (n = 41)

Reference	Authors (Year of Publication)	Quality Rating (AMSTAR-2)[1, 2]
1	McCullough *et al (*2016)	Fair
2	Martin-Biggers *et al (*2014)	Poor
3	Glanz *et al (*2021)	Fair
4	Duriancik & Goff (2015)	Fair
5	Burrows *et al (*2017)	Good
6	Skeer & Ballard (2013)	Poor
7	Dwyer *et al (*2015)	Poor
8	Fulkerson *et al* (2014)	Poor
9	Tosatti *et al (*2017)	Fair
10	Woodruff *et al (*2008)	Fair
11	Robson *et al (*2020)	Good
12	Verhage *et al (*2018)	Fair
13	Middleton *et al (*2020)	Good
22	Hammons & Fiese (2011)	Fair
23	Pearson *et al (*2009)	Fair
24	Scaglioni *et al (*2018)	Poor
25	van der Horst *et al (*2017)	Poor
26	Cislak *et al (*2012)	Good
27	Dallacker *et al (*2019)	Fair
28	Dallacker *et al (*2018)	Fair
29	Harrison *et al (*2015)	Fair
30	Rahill *et al (*2020)	Poor
31	Fraser *et al (*2011)	Poor
32	Liu *et al* (2009)	Good
33	Valdés *et al (*2013)	Fair
34	Khandpur *et al (*2014)	Poor
35	Berge *et al (*2009)	Poor
36	Vollmer & Mobley (2013)	Poor
37	Jenkins & Horner (2005)	Poor
38	Titis (2022)	Poor
39	Do Amaral e Melo *et al (*2020)	Fair
40	Krølner *et al (*2011)	Fair
41	Pearson *et al (*2008)	Good
42	Rasmussen *et al (*2006)	Good
43	Smith *et al (*2022)	Fair
44	Avery *et al (*2017)	Fair
45	Bates *et al (*2018)	Fair
46	Psaltopoulou *et al (*2019)	Fair
47	Beckers *et al (*2021)	Good
48	Goldfarb *et al (*2015)	Fair
49	Dolor-Beauroy-Eustache & Mishara (2021)	Fair

[1] Tool to Assess Systematic Reviews (AMSTAR-2)
[2] Categorisation of AMSTAR-2 score: good (4-5 critical flaws); fair (6-8 critical flaws); poor (9-11 critical flaws)
